# Optimisation of a dilute-and-shoot UHPLC-MS method for high-throughput urinary metabolomics

**DOI:** 10.1007/s11306-026-02542-1

**Published:** 2026-11-13

**Authors:** Patricia Kelly, Caroline Kerbiriou, Bernadette White, Konstantinos Gkikas, Kelly Stewart, Gillian Farrell, Konstantinos Gerasimidis, Nicholas J. W. Rattray

**Affiliations:** 1 School of Medicine, University of Glasgow, Glasgow, UK; 2 Strathclyde Institute of Pharmacy and Biomedical Sciences, 161 Cathedral Street, Glasgow, UK; 3 Strathclyde Centre for Molecular Bioscience, 161 Cathedral Street, Glasgow, UK; 4 NHS Lanarkshire, Wishaw, UK

## Abstract

**Introduction:**

Urinary metabolomics using liquid chromatography-mass spectrometry (LC-MS) holds great potential for biomarker discovery and clinical application. Large-scale studies and clinical trials require rapid, scalable methods. However, existing urine extraction and LC-MS approaches lack standardisation and clear recommendations for high-throughput use.

**Objectives:**

To optimise a dilute-and-shoot urinary metabolite extraction and untargeted ultra-high-performance LC-MS (UHPLC-MS) method suitable for large-scale studies, and to demonstrate its performance in a clinical cohort.

**Methods:**

We systematically optimised eight parameters: extraction solvent, dilution factor, chromatographic gradient, injection volume, flow rate, gradient curve factor, full scan automatic gain control (AGC) and MS/MS AGC. Performance was assessed by the number of metabolites detected, peak quality, creatinine performance and peak quality factors (PQFs). The optimised method was applied to 1094 urine samples (862 clinical samples plus QCs) from healthy controls and patients with inflammatory bowel disease (IBD). We compared three normalisation strategies for their effect on data precision: no normalisation, creatinine normalisation and probabilistic quotient normalisation (PQN).

**Results:**

The optimised method used a C18 column in positive ionisation mode. The mobile phases were water and acetonitrile, each with 0.1% formic acid (v/v). Data acquisition took 6.5 min, and the total run time was 10 min including a cleaning step. In the IBD cohort, over 1500 metabolites were putatively annotated in 182.3 h of instrument time. This saved over 91 h compared with the previously used method.

**Conclusions:**

This optimised method provides rapid, scalable untargeted urinary metabolomics suitable for large clinical studies and trials.

**Graphical abstract:**

**Figure:**
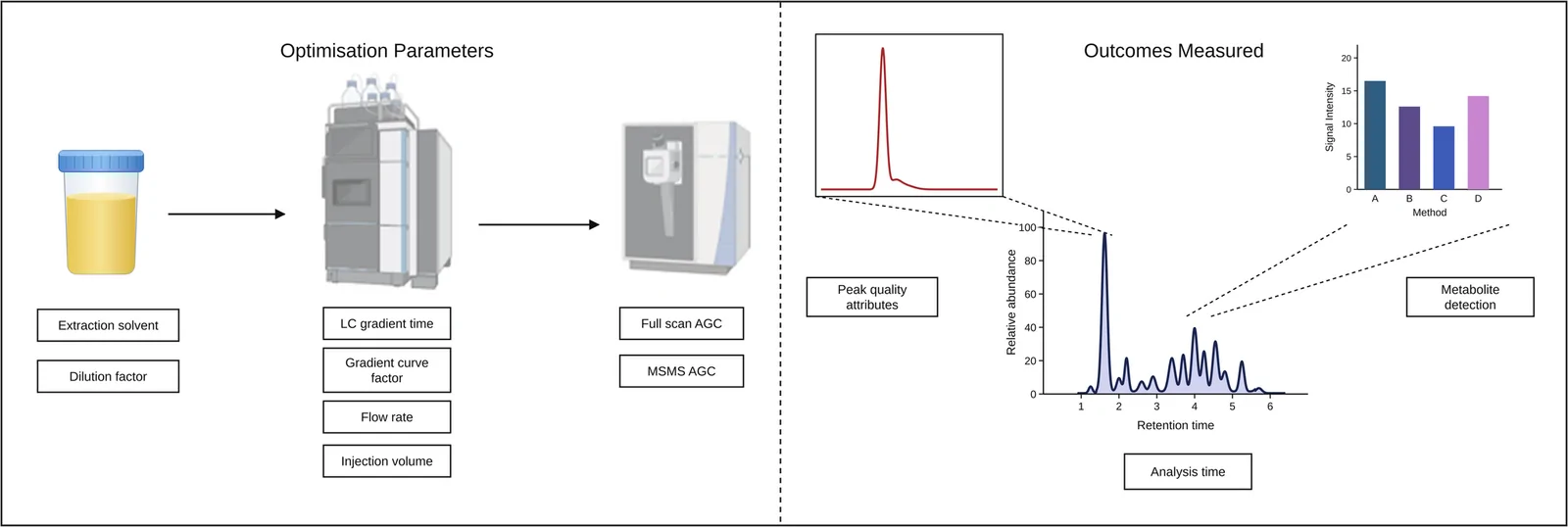

**Supplementary Information:**

The online version contains supplementary material available at https://doi.org/10.1007/s11306-026-02542-1.

## Introduction

Urine is a valuable sample biomatrix for disease research due to the ease and non-invasive nature of collection, making it preferable for large-scale studies (Thomas et al., 2010). The large quantity of urine produced by humans allows for multiple tests to be conducted on the same sample, thereby enabling easier replication and validation and ensuring data consistency across experimental techniques. Furthermore, due to the frequent production of urine, longitudinal studies can be performed more easily, for example to monitor response to treatment or disease progression over time (Hon, 2022). Urine contains a diverse range of biological molecules which reflect metabolic and physiological changes and can therefore act as biomarkers for a multitude of diseases. Urinary biomarkers have been successfully established for a number of diseases and have had significant impacts on clinical care. For example, urinary albumin is a recognised biomarker of kidney damage (Lezaic and Patel, 2015), with the albumin-to-creatinine ratio (ACR) routinely used to diagnose and monitor disease such as chronic kidney disease (CKD) (Chen et al., 2019). Urinary biomarkers have also shown great promise in cancer research, with prostate cancer antigen 3 (PCA3) now used as a biomarker for the diagnosis of prostate cancer (Merola et al., 2015; Opoku Mensah et al., 2022), and in many other fields including metabolic disease (Kabasawa et al., 2022) and neurology research (Duggins-Warf et al., 2023). Among the approaches used for biomarker discovery, metabolomics, the study of small molecules and products of metabolism, has more recently emerged as a tool with great potential to give unique disease insights. 2-hydroxyglutarate, for example, is used as a metabolite biomarker for certain types of brain tumour, particularly gliomas (Fathi et al., 2016, Riviere-Cazaux et al., 2023) and acute myeloid leukaemia (Wang et al., 2013).

The identification of urinary biomarkers and their translation into clinical use has been enabled by advances in the analytical technologies that are used to detect biomarkers. Liquid-chromatography mass spectrometry (LC-MS) is a powerful analytical tool for biomarker discovery and disease mechanism studies, particularly through untargeted approaches, due to its high sensitivity and selectivity and ability to analyse a comprehensive range of chemical molecules found in urine (Joshi et al., 2024; Duijl et al., 2022). Large-scale clinical studies routinely utilise untargeted LC-MS approaches for the identification, validation, and clinical application of biomarkers. The use of a method which both maximises chemical coverage and minimises analysis time and sample deterioration, particularly for the analysis of sensitive metabolites, is therefore vital for application to larger studies.

The pipeline of urinary metabolomics analysis involves several steps, including metabolite extraction, UHPLC-MS data acquisition, and data analysis. Optimisation of the associated method parameters is crucial for accurately detecting the metabolites of interest for the specific aims of a study, which is particularly important in untargeted metabolomics studies where the aim is to maximise coverage of the large range of chemically diverse compounds found in complex biofluids such as urine. Crucially, for large scale metabolomics studies, fast sample preparation and analysis is essential to prevent sample degradation. A rapid extraction and LC-MS method for urinary metabolite detection is therefore optimal for large scale studies to reduce the overall run time and therefore minimise sample degradation across the study duration.

The dilute-and-shoot urine extraction method, which involves diluting the collected sample with a chosen solvent and directly injecting it into the LC-MS system (Zhao et al., 2022; deventer, 2013), offers numerous benefits in large-scale clinical studies, where the focus is on high-throughput and cost-effective analysis of a large number of samples (Gracia-Marín et al., 2024). In comparison to alternative methods, including solid phase extraction (SPE), advantages of the dilute-and-shoot method include a simplified workflow with a reduced processing time, reduced staff time, minimal sample handling and sample loss, and ease of automation and scalability (Tamama, 2023; Castilla-Fernández et al., 2022). As the need for large-scale clinical trials investigating urinary metabolomics grows, the dilute-and-shoot approach will remain essential in biomarker discovery and monitoring. It is therefore vital to work towards the standardisation of an optimal LC-MS method using the dilute-and-shoot extraction approach.

In this investigation, we aimed to optimise an untargeted UHPLC-MS method for urinary metabolomics analysis for large-scale studies while reserving the simplicity of the dilute-and-shoot method. Parameter optimisation was performed across stages of the analysis pipeline, including metabolite extraction, LC, and MS analysis to maximise metabolite identification in urine samples with a reduced preparation and analysis time. To demonstrate the efficiency and applicability of the optimised method, 1094 urine samples from inflammatory bowel disease (IBD) patients and healthy controls were analysed.

## Experimental

### Ethics statement

All participants and their carers provided written informed consent. Approval for the iPENS and CD-TREAT clinical trial studies were granted by the West of Scotland Research Ethics Committee (REC reference: 17/WS/0119, 19/WS/0163, respectively) and NHS Research and Development office. For ENIP the original approval was granted by the College of Medical, Veterinary and Life Sciences Ethics Committee, University of Glasgow (Project number: 200220086). All studies were registered on https://clinicaltrials.gov (identifier NCT number: IPENS, NCT04225689; CD-TREAT, NCT03171246; ENIP, NCT06828094).

### Study design

Individual parameter optimisation was carried out sequentially to improve the performance of the overall method across the experimental pipeline, including the sample preparation protocol and UHPLC-MS method. The best performing parameters were selected using multiple outcome measures, including peak quality attributes, analysis time, and metabolite detection.

### Human urine sample collection

Urine samples were collected from healthy individuals and IBD patients and stored at −80 °C until processing. For the clinical study and application, 1094 urine samples were collected and analysed using the optimised method.

### Chemicals and reagents

LC-MS grade water (H_2_O) and acetonitrile (ACN) were purchased from Fisher Scientific (Geel, Belgium). LC-MS grade formic acid was purchased from Thermo Scientific (Prague, Czech Republic).

### Urine sample preparation

Urine samples were aliquoted into a 96-well plate and diluted with solvent at differing ratios according to the method optimisation stage. The samples were then mixed with a pipette to ensure solvent distribution, and the plate was submitted for UHPLC-MS analysis. ^15^N_2_-labelled tryptophan was used as an internal standard for assessing instrument stability and experimental conditions.

### Untargeted LC-MS metabolite measurement

Untargeted metabolomic analysis was performed on an ultra-high performance liquid chromatography (UHPLC) system (ThermoFisher Scientific) coupled to an Orbitrap Exploris 240 (ThermoFisher Scientific) mass spectrometer. Chromatographic separation was performed on a Vanquish Accucore C18 + UHPLC analytical column (ThermoScientific, 100 mm × 2.1 mm, 2.6 µM). Mobile phase A was composed of 99.9% water + 0.1% formic acid and mobile B was composed of 99.9% ACN + 0.1% formic acid. Electrospray ionisation (ESI) was used as the ionisation method in positive mode (3900 V). The elution gradient used can be found in Supplementary Information Table S1. The source-dependent parameters were operated under the following conditions: sheath gas, 40 Arb; auxiliary gas, 10 Arb; sweep gas, 1 Arb; ion transfer tube temperature, 300 °C; vaporiser temperature, 280 °C. Instrument calibration was performed using Pierce^™^ FlexMix^™^ calibration solution (Thermo Scientific) and ran under vendor recommended settings. MS data collection was performed in data dependent acquisition mode (DDA) to give putative metabolite identification at MSI level 2.

### Feature annotation and metabolite identification

Feature annotation and metabolite identification at MSI Level 2 were performed using Compound Discoverer version 3.3 and metabolites were matched to *mz*Cloud (Advanced Mass Spectral Database, 2025) and ChemSpider (Chemistry, 2025) databases. Data processing involved feature filtration according to pre-defined criteria (Supplementary Information Table S1) and peak quality factor (PQF) quantification was subsequently performed.

### Statistical analysis

Chromatographic data processing and visualisation was carried out using FreeStyle software (Thermo Fisher Scientific). Mass spectrometry data processing and analysis was performed using Compound Discoverer software 3.2 (Thermo Fisher Scientific, Waltham, MA, USA), with processing settings displayed in Supplementary Information Table S2. Statistical analysis was additionally performed using Prism software 10.4.1.

## Results and discussion

Optimisation of the sample preparation protocol and UHPLC-MS method focused on eight key parameters (highlighted in Table 1) to enhance the overall performance and decrease the analysis time of the method. This was carried out sequentially, by firstly optimising the sample preparation method, followed by the liquid chromatography parameters, and finishing with systematic adjustments to mass spectrometry settings. Together, this study analyses sixty parameter comparisons via LC-MS which were analysed in triplicate in Compound Discoverer to make a total of 180 comparisons (Table 1).

**Table 1:** Overall summary of LC-MS protocol parameter optimisation. AGC, automatic gain control

Protocol section	Parameter	Number of parameter settings tested
Metabolite Extraction	Extraction solvent	10
	Dilution factor	4
Liquid Chromatography	Chromatography gradient time	2
	Injection volume	4
	Flow rate	5
	Gradient curve	3
Mass Spectrometry	Full scan AGC	16
	MSMS scan AGC	16

**Fig. 1:**
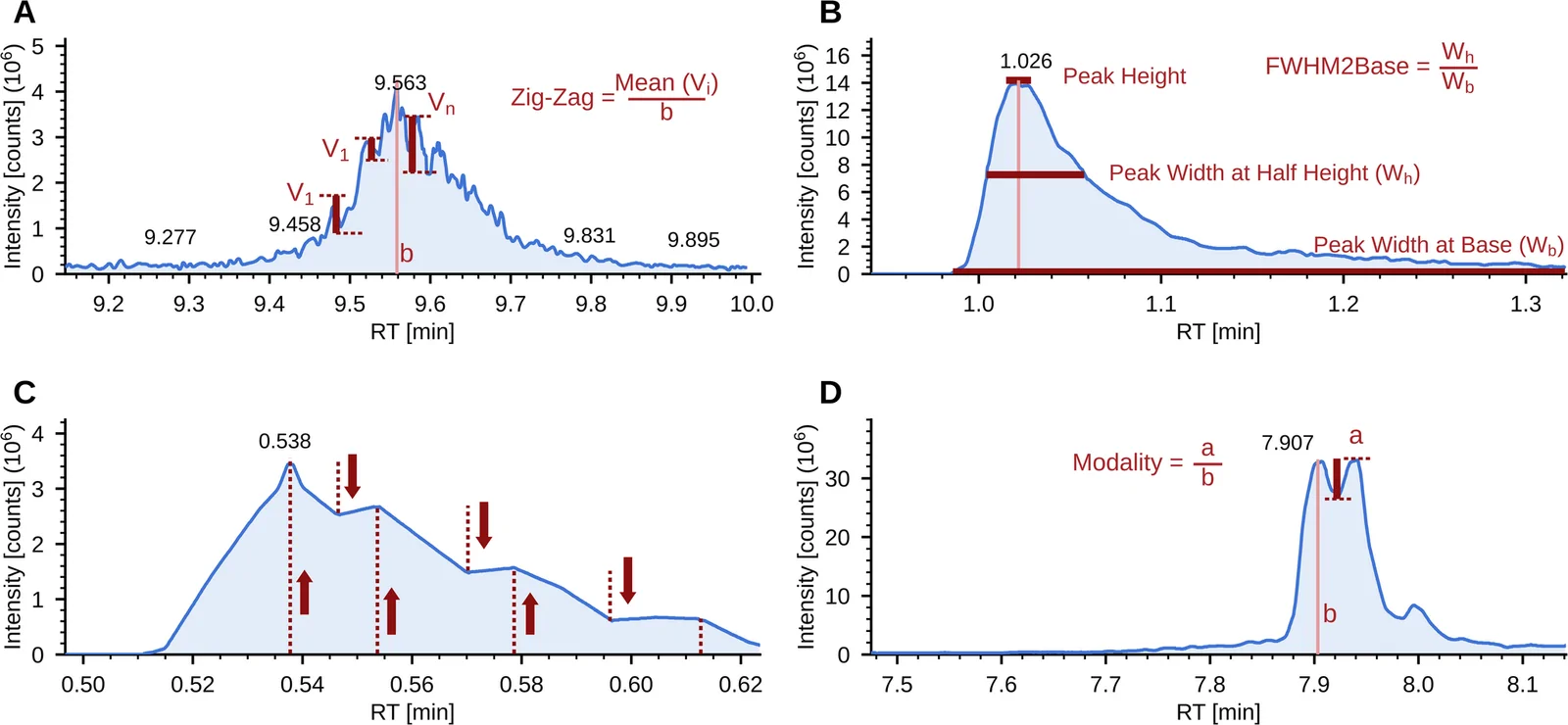
Peak quality factor (PQF) metric description. The **A** zig-zag quality factor, **B** FWHM2Base, **C** jaggedness, and **D** modality quality factors are described, using example peaks from acquired data (Scientific, 2023)

The dilute-and-shoot approach was chosen as no extraction or clean-up step is involved, thus there is no extraction-recovery term to determine; and the absence of analyte loss during minimal sample handling is a recognised advantage of the approach (Tamama, 2023; Castilla-Fernández et al., 2022). The primary metrics used to evaluate parameter performance were the number of metabolites detected after post-acquisition filtration processing and their peak ratings. Additionally, the peak performance of creatinine was evaluated as a metric for evaluating optimisation parameters. Creatinine is a well-characterised stable and endogenous marker present in all urine samples that is commonly used as a reference standard for data normalisation (Hou et al., 2012). Evaluation of its peak performance therefore aids in the assessment of the UHPLC-MS method. Peak quality factors (PQFs) were used to assess peak performance, including the zig-zag index, full width at half maximum to base width (FWHM2Base), jaggedness, and modality factors of individual peaks. Visual representation of each of the PQFs evaluated is provided in Fig. 1, and an overview of outcome parameters is provided in Supplementary Information Table S3. For all PQF settings, a low/zero value refers to a high-quality peak profile, whereas a higher value suggests a poorer peak profile.

### Sample preparation optimisation

Sample preparation is a key determinant of a metabolomics experiment. The ‘dilute-and-shoot’ method was chosen as the urine preparation protocol due to the ease and speed of the preparation protocol, which is a priority for large scale clinical studies. The solvent used for urine metabolite extraction, and the dilution factor were investigated in the present study.

#### Extraction solvent

**Fig. 2:**
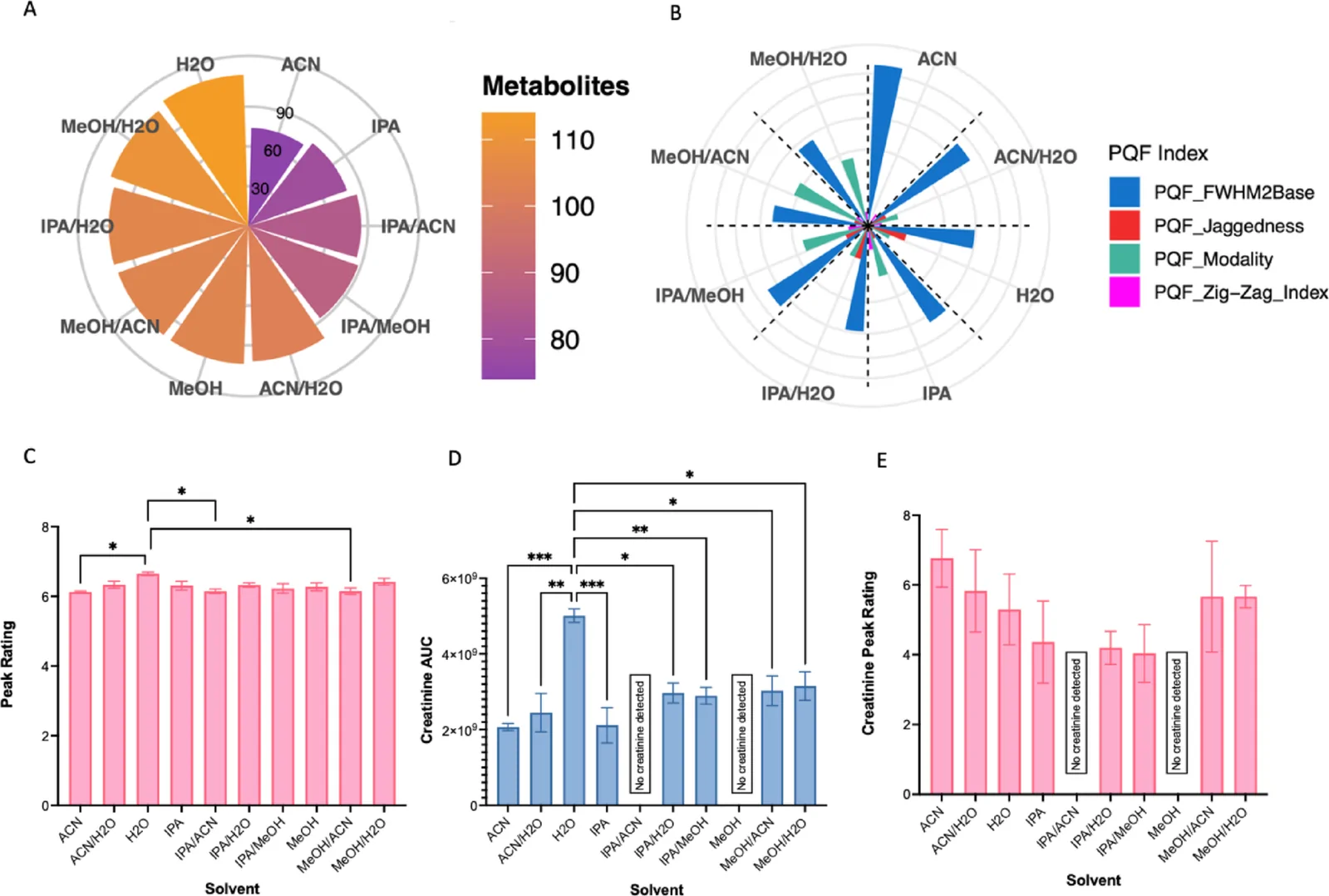
The effect of the extraction solvent on untargeted urinary metabolomics. Outcomes were assessed by **A** the number of metabolites detected, **B** the peak performance of creatinine, as measured by the quantification of peak quality factors (PQF)s, Zigzag, FWHM2Base, Jaggedness, and Modality indices, **C** average peak rating of all metabolites, **D** the area under the curve (AUC) of the detected creatinine peak and **E** the associated creatinine peak rating. *Creatinine was not detected when IPA/H_2_O or MeOH were used as the extraction solvents

For untargeted LC-MS analysis of urine samples using the ‘dilute-and-shoot’ approach, the choice of solvent is crucial to ensure maximal metabolite detection of a wide range of chemical classes. Here, ten solvents/solvent combinations were tested to select the best performing solvent. This was first assessed by analysis of the chromatography of each of the solvents (Supplementary Information Figure S1) and further method performance was measured by quantifying the total number of metabolites detected, their peak rating, and assessment of creatinine as a reference standard (Fig. 2).

Visual inspection of the solvent chromatograms revealed that H_2_O was the only solvent showing successful resolution of peaks between 0.5 and 2.5 min in the method. It was also shown that a 100% H_2_O solvent extraction revealed the highest number of detected metabolites in comparison to all other solvents tested (114), with a significant increase notable when compared to five of the other solvents tested. A significantly higher average peak rating of all metabolites was additionally observed in comparison to three of the other solvents tested. When looking at the values of the creatinine peak PQFs of the different solvents/solvent combinations, there were varied results, however; no significant differences were observed between any of the groups. It can be noted that creatinine was not detected when IPA/H_2_O or MeOH were used as the extraction solvents and therefore PQF analysis was unable to be carried out for these groups. When selecting high quality data in regard to PQF analysis, lower PQF values (e.g., PQF index = 1) correlate to higher quality peaks and higher PQF values (e.g., PQF = 10) correlate to lower quality peaks. With the exception of peak jaggedness, H_2_O showed low/zero creatinine PQF values, indicating a high-quality peak shape and resolution. Parameter data is displayed in more detail in Supplementary Information Figure S2. As demonstrated by an optimal extraction performance and peak performance, H_2_O was therefore selected as the solvent for untargeted LC-MS analysis, and further parameter optimisation was subsequently performed using a H_2_O as the extraction solvent. This is in alignment with other LC-MS methods that have also used a H_2_O extraction for a variety of applications of urinary metabolomics (Zhao et al., 2022; Cao et al., 2015). While it can be noted that there are some studies which suggest that other solvents, for example the use of 25/75 H_2_O/ACN (Hemmer et al., 2024) result in a greater number of metabolite features extracted, sample preparation and treatment prior to LC-MS analysis differs between these studies, and therefore we highlight that the H_2_O extraction was found to be optimal for a dilute-and-shoot preparation method.

#### Dilution factor

The dilution factor used for the H_2_O extraction was subsequently optimised, with the aim of simultaneously maintaining detection of low abundance metabolites, preventing instrument overload, and minimising matrix effects. Supplementary Information Figure S3 displays the peak performance characteristics that were obtained during optimisation.

The number of metabolites detected was significantly higher when a 1:1 dilution was used in comparison to both a 1:5 and a 1:10 dilution (Supplementary Information Figure S4). Results additionally showed that there were no significant differences observed in the average peak rating between the different dilution factors tested. The AUC for the creatinine peak was significantly highest for the 1:1 dilution in comparison to all other parameters tested, with a decreasing trend observed as the dilution factor increased. However, the opposite trend was observed for the peak rating of creatinine, where we see the highest average peak rating for the highest dilution factor used. Creatinine PQF analysis showed no significant differences between the dilution factors for FWHM2Base, and only a 1:1 urine: H_2_O ratio gave a non-zero value for jaggedness and modality factors, indicating a lower peak quality. The dilute-and-shoot approach mitigates matrix effects through dilution, and the dilution factor was optimised in part to minimise matrix interference (Sect. 3.1.2, Figure S3). A formal post-column-infusion matrix-factor determination was beyond the scope of this untargeted optimisation study; we note the reproducibility of the internal standard within matrix-containing QC injections as supporting evidence of stable matrix behaviour, and have added this as a limitation.

### Liquid chromatography parameter optimisation

Following sample preparation, the urine samples were subjected to separation by liquid chromatography, which required the selection of several settings. The chromatography run time, flow rate, and injection volume were individually optimised to define an effective method for untargeted urine metabolomics studies.

#### Chromatography gradient time

A short chromatography method, without significantly compromising metabolite detection, is desirable for use in large-scale clinical studies by enabling high-throughput analysis. In this study, we aimed to reduce the run time of UHPLC-MS urine analysis by shortening the time for chromatographic separation.

The chromatography was optimised to give a method with a data collection time of 6.5 min (70–1000 scan range), with a 3.5-minute cleaning step to give a total run time of 10 min. The gradient was designed to maximise metabolite detection throughout the analysis time and avoid empty chromatographic space. It is essential during method optimisation to ensure that a shorter chromatographic analysis time doesn’t significantly sacrifice metabolite detection, and therefore the two methods were compared regarding the number of metabolites identified by each method and their PQFs (Supplementary Information Figure S5). There was no significant difference in the number of metabolites detected or the peak quality between the two methods, confirming that the 6.5-minute method provides a shorter overall analysis time without sacrificing metabolite detection, which is an essential consideration for clinical metabolomics studies. The AUC for creatinine was shown to be significantly lower in the optimised method, however the inverse relationship was observed for the peak quality of creatinine, although the differential peak quality did not reach significance. No significant differences were observed for the PQF rating of either FWHM2Base, jaggedness, or modality, however the optimised method gave a zero value for the latter, indicating improved peak shape of the new shorter method.

#### Injection volume

The injection volume used in an LC-MS run is a critical method parameter directly affecting the analytical performance of an experiment. The specific amount of analyte injected into the column affects the peak shape and resolution of the data obtained, and a balance is required to determine a large enough signal intensity for optimal detection of metabolites, including low-abundance metabolites, and preventing column overload. A variety of injection volumes ranging from 0.5 to 5µL were compared in the current study to determine the optimal volume for untargeted urine metabolomics using the Orbitrap-240 system, as shown in Supplementary Information Figure S6.

Examination of method parameters revealed that an injection volume of 5 µL resulted in the greatest number of metabolites detected Supplementary Information Figure S7, with no significant differences in their average peak rating. However, when a 5 µL injection volume was used, the creatinine peak rating was significantly lower than a 1 µL and 2 µL injection volume. While metabolite quantification would initially suggest that a 5 µL injection volume may be optimal for the UHPLC-MS method, the poor peak quality of creatinine, as demonstrated by the peak broadening in the creatinine chromatogram and large creatinine AUC, led to the selection of a 2 µL as the optimal injection volume. This additionally reduces the risk of column overload, which is an important consideration for studies analysing a large number of samples. Several published studies have also utilised an injection volume of 2 µL in urinary mass spectrometric analysis (Scheijen et al., 2012; Wei et al., 2014; Al-Saffar et al., 2013) which support the results obtained from the present analysis.

#### Flow rate

The rate at which the solvent passes through the system also affects chromatographic and ionisation performance and is therefore a crucial parameter to be optimised as part of method development. A range of flow rates were applied to the method with multiple outcomes compared (Supplementary Information Figure S8).

The number of metabolites detected was significantly higher at 0.4 mL/min when compared to all other flow rates investigated (Supplementary Information Figure S9). For the average peak quality of all putatively identified metabolites, an increasing trend was observed, with 0.4 mL/min showing a significantly higher peak quality in comparison to the lower flow rates, but lower when compared to the higher flow rates. When looking into detection quality of creatinine, we can see that 0.4 mL/min shows the highest AUC and a significantly higher peak rating than 0.25 mL/min. The jaggedness was the only PQF that demonstrated differential significance, with 0.25 mL/min showing a higher value than all other flow rates. Due to the significantly increased metabolite detection, high performing average peak and creatinine peak data, and prevention of column overload, 0.4 mL/min was chosen as the optimal flow rate parameter for the current method. This view is supported by a range of studies noting the use of a 0.4 mL/min flow rate for LC-MS methods analysing urine samples (Liu et al., 2016; Leninskii et al., 2023; Ming and Heathcote, 2011).

#### Gradient curve

During the chromatographic separation process, the gradient of the mobile phase is represented by the composition change of solvent A (in this case, water + 0.1% FA) and solvent B (acetonitrile + 0.1% FA) over time. The gradient curve refers to the curve shape parameter, defining how the solvent gradient progresses throughout the method, and the rate of solvent composition change. The value of the gradient curve is therefore important to optimise in an LC-MS method, as it directly impacts the retention time and separation efficiency of detected metabolites. A gradient curve of 3, 5, and 7 were applied to the method and analysed (Supplementary Information Figure S10).

As shown in Figure S11, metabolite detection, peak ratings and peak quality factors did not differ significantly between gradient curves 3, 5 and 7. Curve 7 was selected on the basis of improved chromatographic peak resolution and peak shape observed in the extracted chromatograms (Figure S10). Details of the final chromatography elution gradient, including each of the liquid-chromatography method parameters optimised, are shown in Supplementary Information Table S4.

### Mass spectrometer parameter optimisation

#### Automatic gain control (AGC)

The automatic gain control (AGC) is used for controlling the number of ions that are accumulated during a survey scan in the orbitrap analyser. A higher target value refers to a longer accumulation time and therefore implicates an increased analysis time with improved sensitivity, and vice versa. This accumulation time, also known as transient time, is a critical factor in determining the resolution of the mass analyser. Longer transient times allow more precise measurement of ion oscillations, increasing resolution and enabling more accurate assignment of m/z values to detected ions. However, this sacrifices the cost of throughput, as longer transients reduce the number of spectra required per unit time, linking AGC settings closely with both data quality and acquisition speed. The maximum ion injection time setting controls the maximum time the instrument will spend accumulating ions for each scan, which requires a balance between ensuring enough time for appropriate ion accumulation and speed of overall analysis. Optimisation of the specific AGC target value and maximum ion injection time settings is a powerful tool to control the analytical output and peak parameters. We therefore performed method optimisation of AGC parameters for both full and MSMS scans.

**Fig. 3:**
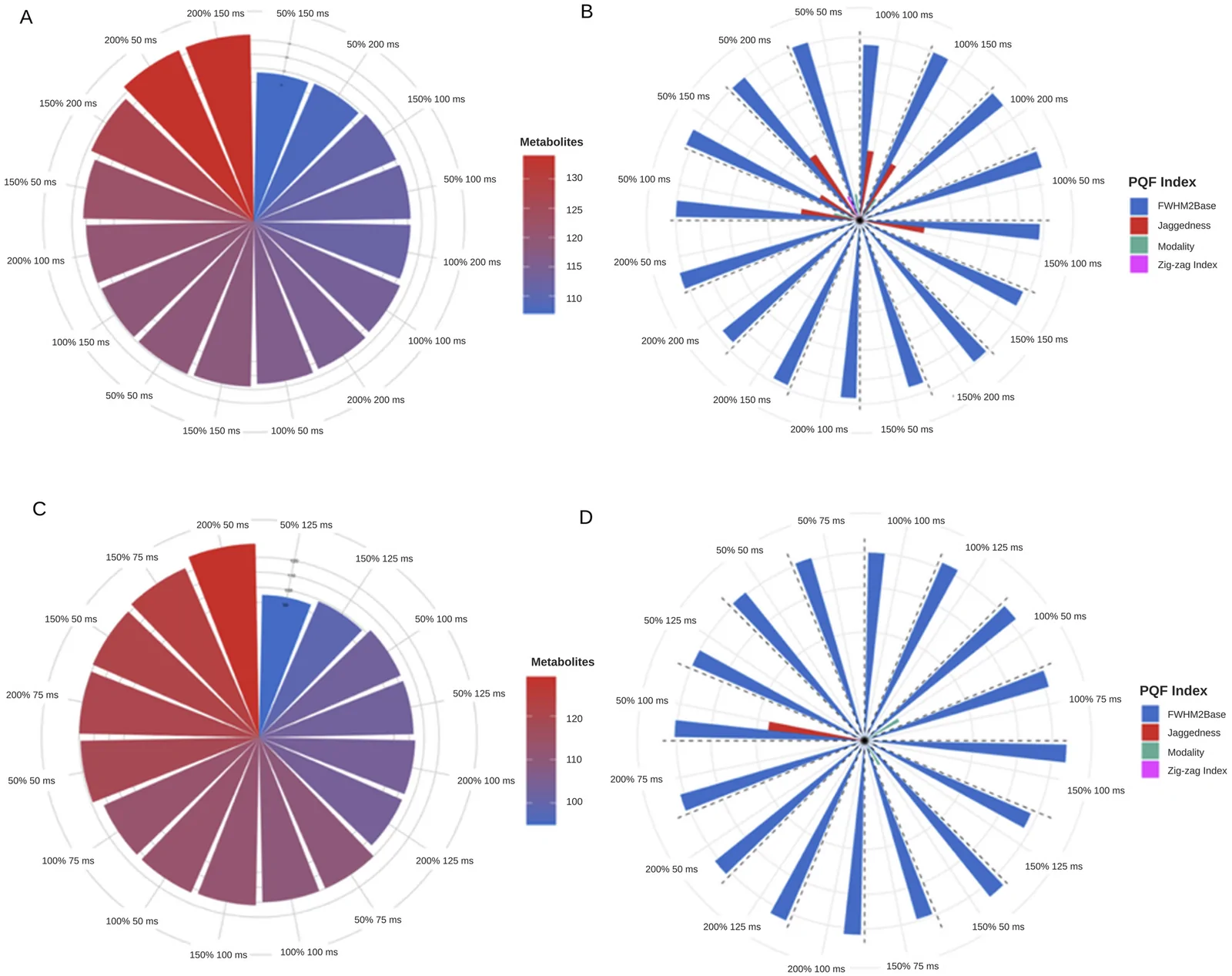
Comparison of AGC parameters and their impact on untargeted urinary metabolomics. Outcomes were assessed by **A** the number of metabolites detected per full scan AGC parameter, **B** the associated peak performance of creatinine, as measured by the quantification of peak quality factors (PQF)s, Zigzag, FWHM2Base, Jaggedness, and Modality indices, **C** the number of metabolites detected per MSMS scan AGC parameter, and **D** peak performance characteristics for the MSMS scan settings. The zig-zag indices are not shown as all parameters tested gave a zero value

The full scan and MSMS AGC parameters were optimised, with all potential setting combinations on the Orbitrap 240 analysed and compared (Fig. 3). This included four AGC target values, expressed as percentage AGC, and four maximum injection time values. Analysis was further extended to optimisation of MSMS AGC parameters, which similarly involved investigation into all potential settings on the Orbitrap 240 (four AGC target values and four maximum injection time values) for the tandem mass spectrometry settings. Detailed parameter analysis of the full scan and MSMS AGC optimisation are displayed in Supplementary Information Figure S12 and S13.

Notably, no significant differences were observed in the number of metabolites detected, the creatine AUC and peak rating, or PQFs for the full scan AGC analysis, however, we can comment on some data trends. When comparing the four AGC parameters, 50% and 100% target values showed a more variable peak performance when looking at the PQF analysis, which may be explained by insufficient ion accumulation at these lower values. On the other hand, the highest AGC target of 200% resulted in a slight increase in signal intensity but did not significantly improve the number of metabolites detected. Alongside analysis of the chromatograms obtained from each parameter, the decision was made to select an AGC target of 150%, as driven by its peak performance, signal intensity, and metabolite detection. Similarly, the choice of a maximum injection time of 100 ms provided a favourable balance between ion collection and spectral acquisition speed. Overall, a full scan AGC target of 150% with a 100 ms maximum injection time was selected to be the most effective configuration for this method.

**Fig. 4:**
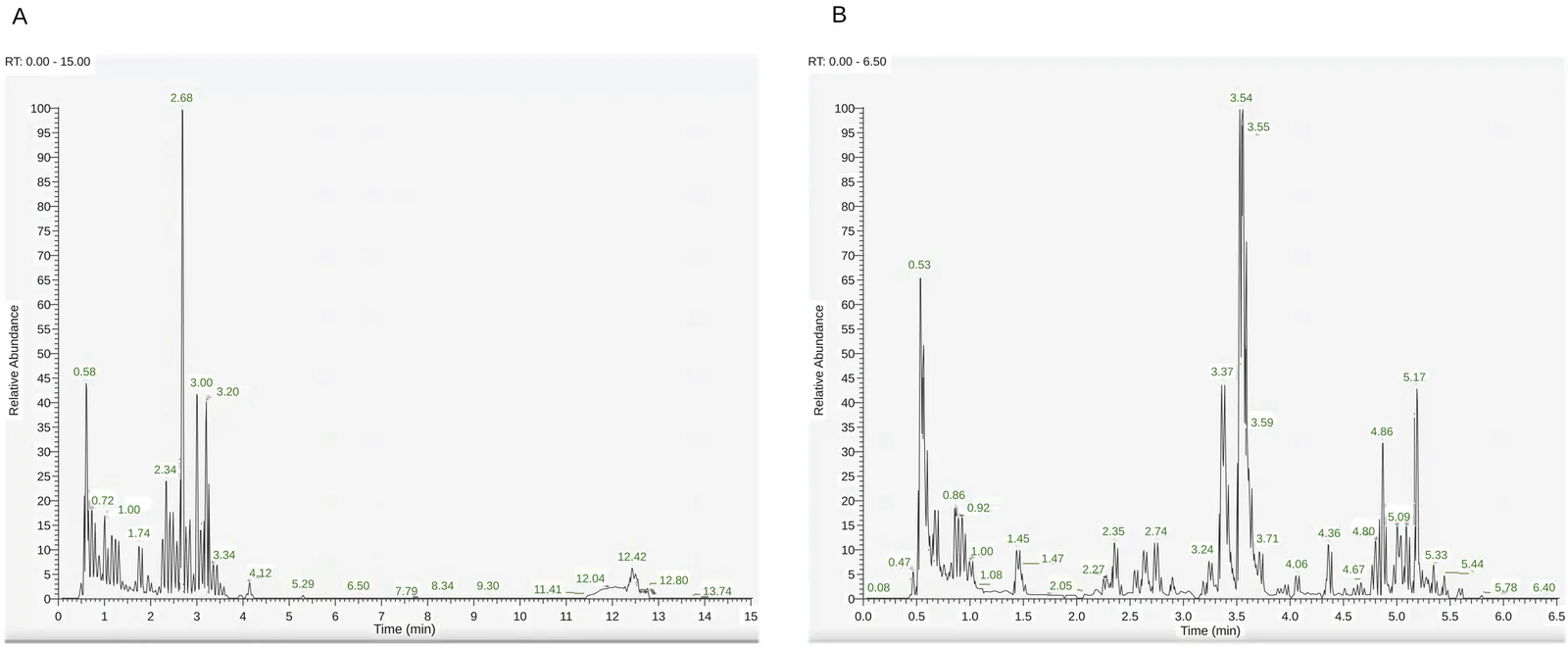
Method optimisation overview. Comparison of **A** the original 15-minute method and **B** shortened 10-minute chromatography gradient

For the MSMS scan analysis, it was observed that increasing the AGC target above 100% resulted in a slight increase in signal intensity, however it did not significantly enhance the overall number of metabolites detected. This suggests that ion saturation may occur at higher MSMS AGC settings (Hall et al., 2013). A 100% AGC target is therefore suggested for optimal ion accumulation without the increased risk of saturation. A maximum injection speed of 50 ms maintained high PQFs, particularly with respect to the zigzag, jaggedness, and modality metrics which consistently showed zero values. An MSMS AGC target of 100% with a 50 ms maximum injection time was therefore selected to be the most effective configuration, which completed the parameter optimisation to finalise the method. The final optimised method for untargeted urinary UHPLC-MS analysis includes a streamlined and shortened preparation and analysis process with improved metabolite detection and peak performance metrics. A chromatographic overview of the two methods is shown in Fig. 4.

### Application of the method to clinical samples

The optimised method was subsequently applied to a clinical study which analysed 1094 urine samples from healthy individuals and IBD patients, making this the world’s largest study to date investigating urinary metabolomics of IBD. The whole trial consisted of three different patient cohorts: The ENIP (exclusive or partial enteral nutrition in healthy individuals) study investigated the effect of enteral nutrition, a liquid diet used as the primary treatment for patients with Crohn’s disease (Connors et al., 2017), and explored the impact on healthy individuals, and involved the analysis of 107 urine samples. The CD-TREAT (Crohn’s Disease Treatment-with-EATing) diet is a prescriptive diet which recreates Exclusive Enteral Nutrition (EEN) (Svolos et al., 2019). The optimised urine method was applied to investigate disease outcomes and prediction response of CD-TREAT using UHPLC-MS, where 125 urine samples were analysed. The intensive Post Exclusive Enteral Nutrition Study (iPENS) study, a randomised trial to evaluate CD-TREAT diet as a food reintroduction regime in children and young adults with Crohn’s disease, investigated metabolic signatures in different biomatrices including urine. In total, 1344 injections were analysed (1094 clinical urine samples plus 250 pooled QC) throughout the clinical trial using the method presented in this paper, as displayed in Supplementary Information Figure S14.

In comparison to the previous method which had a run time of 15 min and equating to 273.5 h of analysis time, the optimised 10-minute method allowed all samples to be run in 182.3 h, saving a total of over 91 h of LC-MS analysis time throughout the clinical trial and therefore demonstrating an improvement in method throughput, without sacrificing data quality. This has important implications for future large-scale studies, where time of analysis is an essential factor to prevent sample degradation during the duration of sample preparation and analysis. Many existing large scale urinary metabolomics studies to date have used longer chromatography elution gradients. For example, the analysis of urine samples from 348 healthy children and 315 adults characterising age- and sex-dependent metabolic variations used an 18-minute chromatography method (Liu et al., 2022), a 28.5-minute elution gradient was used to identify urinary biomarkers of type 2 diabetes patients (Chen et al., 2018), and a 29-minute elution gradient was used for the comprehensive profiling of the normal human metabolome using 663 urine samples (Xu et al., 2022). This demonstrates the varied application of urinary LC-MS studies and the potential for method standardisation, efficiency improvement, and cost reduction.

### Comparison of urine normalisation strategies for adjusting urine dilution

Normalisation is essential in urinary metabolomics to correct for differential sample dilution, particularly in untargeted LC-MS workflows. Creatinine normalisation and probabilistic quotient normalisation (PQN) are two widely used approaches for adjusting urine data post-acquisition. Creatinine normalisation corrects for dilution based on the concentration of urinary creatinine, while PQN adjusts each sample based on the overall distribution of metabolites relative to quality control (QC) reference samples. In this study, we compared both approaches alongside non-normalised data to evaluate their impact on data quality. To assess the global impact of normalisation on data structure, principal component analysis (PCA) was performed on each dataset. Minimal differences were observed in the PCA plots between the three conditions (Supplementary Figure S15), suggesting that PCA-based evaluation alone is insufficient to differentiate between data quality of the normalisation methods.

**Fig. 5:**
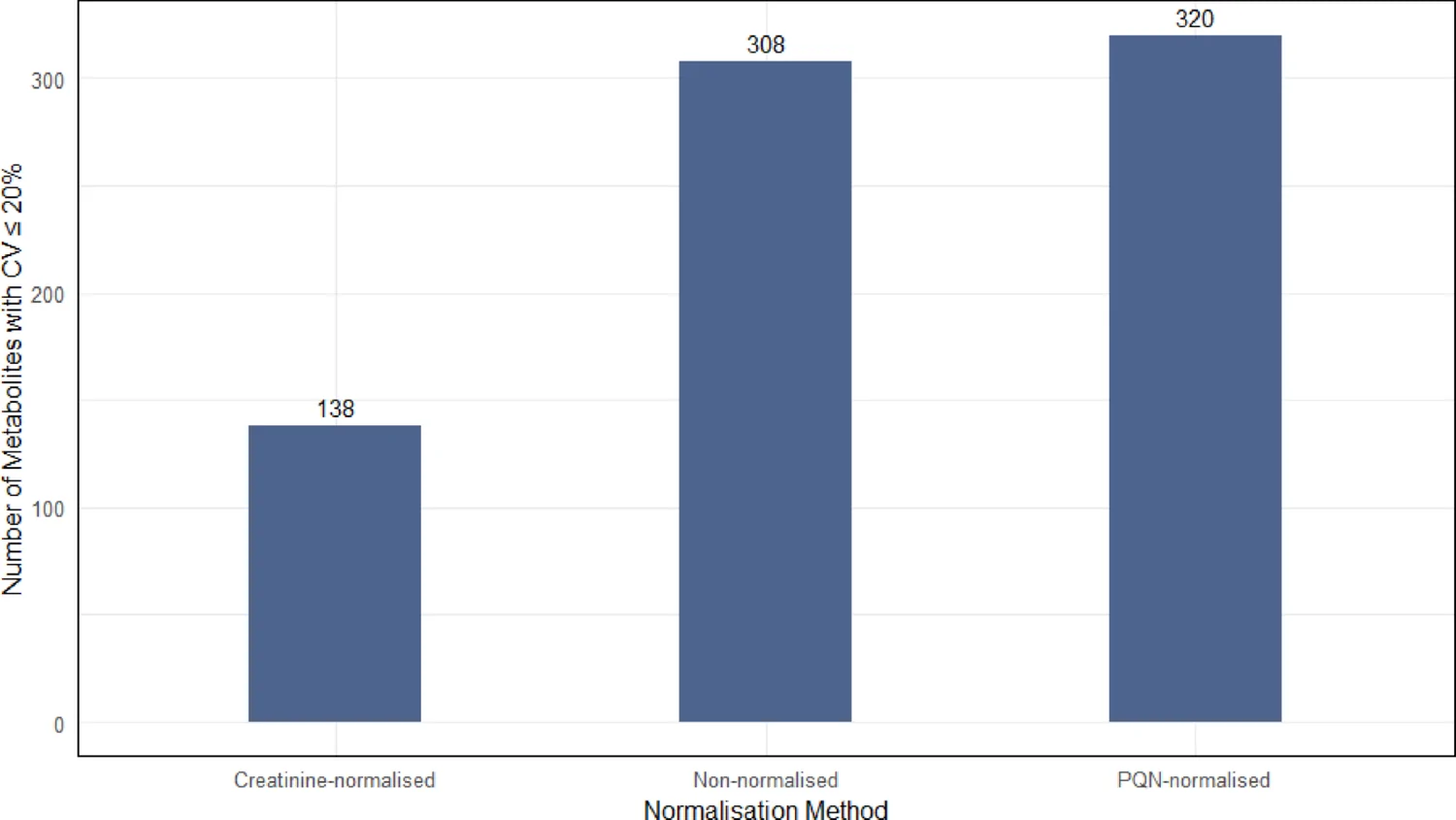
Number of metabolites with coefficient of variation (CV) ≤ 20% across three normalisation methods. The bar plot shows the total number of metabolites meeting a CV threshold of ≤ 20% for non-normalised data, creatinine normalised data, and probabilistic quotient normalisation (PQN) normalised data

The coefficient of variation (CV) was calculated for each metabolite across QC samples to evaluate the precision of each normalisation strategy. A CV threshold of ≤ 20% was applied, and the number of metabolites that met the cut-off using each normalisation method were quantified (Fig. 5). PQN normalisation showed the highest number of metabolites that met this criterion (*n* = 320), followed by the non-normalised data (*n* = 308). In contrast, creatinine normalisation resulted in substantially fewer metabolites below the threshold (*n* = 138). These findings suggest that PQN normalisation offers superior performance in terms of analytical precision as measured by CV whereas creatinine normalisation should not be used owing to poorer results upon comparison to non-normalised data. Detailed CV values for the detected metabolites (MSI level 2) using each of the normalisation methods are provided in Supplementary Information Table S5.

To explain the poor performance of creatinine normalisation, we examined both the per-metabolite CV distributions (Table S5) and the variability of the creatinine signal itself across the clinical cohorts. Relative to non-normalised data, creatinine normalisation increased the CV of the majority of metabolites (median CV inflation of approximately 1.2-fold), whereas PQN reduced the CV of most metabolites. In pooled QC injections the creatinine peak area was highly reproducible (CV of 3.0%), confirming that creatinine is quantified reliably by the method and that matrix interference in the creatinine measurement is not a limiting factor. Independent confirmation comes from the ¹⁵N₂-tryptophan internal standard, which is added at a constant amount to every sample and therefore carries no biological variation: its peak area was stable across the full acquisition with negligible drift and a relative standard deviation of 14.4% (8.4 to 15.0% across cohorts; Figure S16), establishing an analytical variability floor well below the biological variability of creatinine. Across the clinical samples, by contrast, the creatinine signal was markedly more variable (CV of approximately 35% in the iPENS cohort and 29% in CD-TREAT), spanning an approximately ten-fold range between the least and most concentrated samples and a 2.8-fold range across the central 90% of samples (Figure S17). Biological variability in creatinine excretion therefore exceeded its analytical variability by more than ten-fold, and was of similar magnitude in Crohn’s disease and healthy control samples, indicating that it reflects a general property of spot-urine sampling in this population rather than disease status alone.

This variability accounts directly for the observed normalisation performance. Because creatinine normalisation rescales every feature by this single, biologically variable analyte, its excretion variance propagates to all metabolites and inflates their CVs, reducing the number meeting the CV threshold to 138. The variability is consistent with the established dependence of creatinine excretion on age, sex, body composition, renal function and, in these cohorts, dietary intervention (exclusive enteral nutrition and CD-TREAT), which makes creatinine an unstable dilution proxy in this setting. PQN instead derives a sample-specific dilution factor from the median behaviour of the entire feature set relative to QC samples, and is therefore substantially more robust, consistent with the CV distributions observed (Fig. 5).

## Conclusions

The main purpose of this study was to develop a rapid and reliable method for large-scale comprehensive LC-MS metabolic profiling. We present a 10-minute UHPLC-MS method, comprised of a 6.5-minute data collection time and a 3.5-minute cleaning step, based on urine samples extracted using the dilute-and-shoot method. Optimisation of eight individual method parameters were sequentially carried out to provide an overall method which effectively detects a large number of metabolites of high data quality, as determined by a range of peak performance quality factors. Additionally, probabilistic quotient normalisation (PQN) was identified as the most effective post-acquisition strategy for correcting urine dilution, compared to creatinine or no-normalisation. This study successfully investigated the possibility to reduce the overall UHPLC-MS method analysis time for untargeted urinary metabolomics, which has positive implications for high-throughput and large-scale clinical trials, as demonstrated through the application of the optimised method to a global urinary metabolomics study.

## Supplementary Information

Below is the link to the electronic supplementary material.

Supplementary Material 1

## Data Availability

No datasets were generated or analysed during the current study.
