# Supplementary material for: Optimisation of a dilute-and-shoot UHPLC-MS method for high-throughput urinary metabolomics
Source: Metabolomics. 2026 Nov 13;22(6):176. doi: 10.1007/s11306-026-02542-1 (PMC13768220; doi:10.1007/s11306-026-02542-1)
Supplement: Supplementary file 1 [file 11306_2026_2542_MOESM1_ESM.docx]

# **SUPPLEMENTARY INFORMATION**

**Optimisation of a Dilute-and-Shoot UHPLC-MS Method for High-Throughput Urinary Metabolomics**

Patricia Kelly^1^, Caroline Kerbiriou^1^, Bernadette White^1^, Konstantinos Gkikas^1^, Kelly Stewart^1^, Gillian Farrell^2^, Konstantinos Gerasimidis^1^, Nicholas JW Rattray^2+3+4^

1 School of Medicine, University of Glasgow, UK

2 Strathclyde Institute of Pharmacy and Biomedical Sciences, 161 Cathedral Street, Glasgow, UK

3 Strathclyde Centre for Molecular Bioscience, 161 Cathedral Street, Glasgow, UK

4 NHS Lanarkshire

**Table S1**. Compound Discoverer metabolite filter settings.

| Compound Discoverer Filter | Setting |
| --- | --- |
| Name | Is not blank |
| Annotated Δ Mass [ppm] | Is between -5.00 and 5.00 |
| Annotation Source | Has status full match in source Predicted Compositions |
| Annotation Source | Has status full match in source ChemSpider Search |
| MS2 | Is not equal to No MS2 |

**Table S2**. Compound Discoverer workflow settings.

| Workflow Node | Workflow Parameter | Workflow Information |
| --- | --- | --- |
| Input files |  | .raw data |
| Select Spectra | Spectrum Properties Filter | Lower RT limit: 0  Upper RT limit: 0 |
|  | Scan Event Filters | Polarity Mode: Is + |
| Align Retention Times | General Settings | Alignment Model: Adaptive curve  Maximum Shift [min]: 0.3  Mass Tolerance: 2 ppm |
| Detect Compounds | General Settings | Mass Tolerance: 2 ppm  Intensity Tolerance [%]: 30  S/N Threshold: 5  Min. Peak Intensity: 2 000 000  Ions: [2M + FA + H]-1; [2M + H]+1; [2M + K]+1; [2M + Na]+1; [2M - H]-1; [M + 2H]+2; [M + Cl]-1; [M + FA - H]-1; [M + H]+1; [M + H + K]+2; 2M + H + MeOH]+1; [M + H + Na]+2; [M + H – H_2_O]+1; [M + K]+1; [M + Na]+1; [M - 2H]-2; [M + 2H + K]-1; [M -H]-1; [M -H_2_O]-1  Min. Element Counts: C H  Max. Element Counts: C90 H190 Br3 Cl4 K2 N10 Na2 O15 P5 S5 |
| Group Compounds | Compound Consolidation | Mass Tolerance: 2 ppm  RT Tolerance [min]: 0.2 |
|  | Fragment Data Selection | Preferred Ions: [M - H] +1; [M- H] -1 |
| Search mzCloud | General Settings | Compound Classes: Endogenous Metabolites, Excipients/ Additives/ Colourants, Extractables/ Leachables, Natural Products/ Medicines, Natural Toxins, Personal Care Products/ Cosmetics, Small Molecule Chemicals, Steroids/ Vitamins/ Hormones, Therapeutic/ Prescription Drugs  Precursor Mass Tolerance: 10 ppm  FT Fragment Mass Tolerance: 10 ppm  Library: Autoprocessed, Reference  Post. Processing: Recalibrated  Annotation Matching. Fragments: True |
|  | DDA Search | Identity Search: Cosine  Match Activation Type: True  Match Activation Energy: Match with Tolerance  Activation Energy: 20  Apply Intensity Threshold: True  Similarity Search: None  Match Factor Threshold: 60 |
|  | DIA Search | Use DIA Scans: False  Max. Isolation Width [Da]: 500  Match Activation Type: False  Match Activation Energy: Any  Activation Energy Tolerance: 100  Apply Intensity Threshold: False  Match Factor Threshold: 20 |
| Predict Compositions | Prediction Settings | Mass Tolerance: 2 ppm  Min. Element Counts: C. H  Max. Element Counts: C90 H190 Br3 Cl4 K2 N10 Na2 O15 P5 S5  Min. RDBE: 0  Max. RDBE: 40  Min. H/C: 0.1  Max. H/C: 4  Max.# Candidates: 10  Max.# Internal Candidates: 200 |
|  | Pattern Matching | Intensity Tolerance [%]: 30  Intensity Threshold [%]: 0.1  S/N Threshold: 3  Min. Spectral Fit [%]: 30  Min. Pattern Cov. [%]: 90  Use Dynamic Recalibration: True |
|  | Fragments Matching | Use Fragments: True  Mass Tolerance: 2 ppm  S/N Threshold: 5 |
| Map to Metabolika Pathways | Search Settings | Metabolika pathways: All  Search Mode: By Formula or Mass |
|  | By Mass Search Settings | Mass Tolerance: 2 ppm |
|  | By Formula Search Settings | Max. # of Predicted Compositions to be searched per Compound: 3 |
|  | Display Settings | Max. # of Pathways in ‘Pathways’ column: 20 |
| Apply mzLogic | General Settings | FT Fragment Mass Tolerance: 10 ppm  IT Fragment Mass Tolerance: 0.4 Da  Max. # Compounds: 0  Max. # mzCloud Similarity Results to consider per Compound: 10  Match Factor Threshold: 30 |
| Assign Compound Annotations | General Settings | Mass Tolerance: 2 ppm |
|  | Data Sources | Data source #1: mzCloud Search  Data source #2: Predicted Compositions  Data source #3: MassList Search  Data source #4: ChemSpider Search  Data source #5: Metabolika Search |
|  | Sorting Rules | Use mzLogic: True  Use Spectral Distance: True  SFit Threshold: 20  SFit Range: 20 |
| Fill Gaps | General Settings | Mass Tolerance: 2 ppm  S/N Threshold: 5  Use Real Peak Detection: True |
| Apply QC Correction | General Settings | Min. QC Coverage [%]: 30  Max. QC Area. RSD [%]: 30  Max. Corrected QC Area RSD [%]: 25  Max. # Files Between QC Files: 15 |
| Mark Background Compounds | General Settings | Max. Sample/ Blank: 5  Max. Blank/ Sample: 0  Hide Background: True |
| Differential Analysis | General Settings | Log10 Transform Values: True |

**Table S3**. Peak Quality Factor Descriptions.

| Peak Quality Factor (PQF) | Description |
| --- | --- |
| Zig-zag | The zig-zag index measures the vertical peak smoothness, which is quantified by calculating the number and magnitude of vertical fluctuations, i.e., zig-zags in the signal intensity profile. |
| FWHM2Base | The FWHM2Base is defined as the ratio of the FWHM to the full width at baseline, thereby providing a metric for quantifying peak symmetry, resolution, and tailing. |
| Jaggedness | A quantification of the smoothness of a peak as measured by the irregularity or variability of the signal intensity. |
| Modality | The modality index indicates the number of peak apexs and infers the peak resolution and elution properties. |

**Figure S1.** Comparison of chromatograms obtained from untargeted LC-MS analysis using different solvent systems for method optimisation. Each colour represents a solvent used for extraction with signal intensities shown. The colours are represented by the following solvents: black – Acetonitrile (1.90E9), red - Acetonitrile/ H_2_O (2.5E9), blue – H_2_O (2.3E9), orange – IPA (1.81E9), pink – IPA/ ACN (2.22E9), green – IPA/ H_2_O (2.07E9), brown – IPA/MeOH (2.05E9), light blue – MeOH (2.39E9), grey – MeOH/ ACN (2.44E9), purple – MeOH/H_2_O (2.22E9).

**Figure S2.** The effect of the extraction solvent on untargeted urinary metabolomics. Outcomes were assessed by **(A)** the number of metabolites detected, **(B)** their average peak rating, **(C)** the area under the curve (AUC) of the detected creatinine peak and **(D)** the associated peak rating. The peak performance creatinine of creatinine further evaluated, as measured by the quantification of peak quality factors (PQF)s, **(E)** Zigzag, **(F)** FWHM2Base, **(G)** Jaggedness, and **(H)** Modality indices. *Creatinine was not detected when IPA/H_2_O or MeOH were used as the extraction solvents.

**Figure S3.** Comparison of chromatograms obtained from untargeted LC-MS analysis using different extraction solvent dilution factors for method optimisation. Each colour represents a solvent used for extraction with signal intensities shown. The colours are represented by the following solvents: black – dilution factor 1 (1.7E9), red – dilution factor 2 (2.21E9), green – dilution facctor 5 (1.7E9), blue – dilution factor 10 (1.34E9).

**Figure S4.** The effect of the dilution factor on untargeted urinary metabolomics. Outcomes were assessed by **(A)** chromatographic visualisation, **(B)** the number of metabolites detected, **(C)** their average peak rating, **(D)** the area under the curve (AUC) of the detected creatinine peak and **(E)** the associated peak rating. The peak performance of creatinine was further evaluated, as measured by the quantification of peak quality factors (PQF)s, **(F)** FWHM2Base, **(G)** Jaggedness, and **(H)** Modality indices. The chromatogram presented in (A) represents the optimised method parameter selected for the method. The zig-zag indices are not shown as all parameters tested gave a zero value.

**Figure S5.** The effect of chromatography gradient elution time. The effects of the chromatography analysis time on untargeted urinary metabolomics were assessed by **(A)** the number of metabolites detected, **(B)** their average peak rating, **(C)** the area under the curve (AUC) of the detected creatinine peak and **(D)** the associated peak rating. The peak performance creatinine of creatinine further evaluated, as measured by the quantification of peak quality factors (PQF)s, **(E)** FWHM2Base, **(F)** Jaggedness, and **(G)** Modality indices. The zig-zag indices are not shown as all parameters tested gave a zero value.

**Figure S6.** Comparison of chromatograms obtained from untargeted LC-MS analysis using different injection volumes for method optimisation. Each colour represents a solvent used for extraction with signal intensities shown. The colours are represented by the following solvents: black – 0.5 (5.09E8), red – 1 (8.3E8), green – 2 (1.31E9), blue – 5 (2.07E9).

**Figure S7.** The effect of injection volume on untargeted urinary metabolomics. Outcomes were assessed by **(A)** chromatographic visualisation, **(B)** the number of metabolites detected, **(C)** their average peak rating, **(D)** the area under the curve (AUC) of the detected creatinine peak and **(E)** the associated peak rating. The peak performance creatinine of creatinine further evaluated, as measured by **(F)** the FWHM2Base. The chromatogram presented in (A) represents the optimised method parameter selected for the method. The zigzag, jaggedness, and modality indices are not shown as all parameters tested gave a zero value.

**Figure S8.** Comparison of chromatograms obtained from untargeted LC-MS analysis using different flow rates for method optimisation. Each colour represents a solvent used for extraction with signal intensities shown. The colours are represented by the following solvents: black – 0.25 mL/min (1.86E9), red – 0.3 mL/min (2.42E9), green – 0.4 mL/min (2.31E9), blue – 0.5 mL/min (2.35E9), pink – 0.6 mL/min (2.30E9).

**Figure S9.** The impact of flow rate on untargeted urinary metabolomics. Outcomes were assessed by **(A)** chromatographic visualisation, **(B)** the number of metabolites detected, **(C)** their average peak rating, **(D)** the area under the curve (AUC) of the detected creatinine peak and **(E)** the associated peak rating. The peak performance of creatinine was further evaluated, as measured by the quantification of peak quality factors (PQF)s, **(F)** Zigzag, **(G)** FWHM2Base, **(H)** Jaggedness, and **(I)** Modality indices. The chromatogram presented in (A) represents the optimised method parameter selected for the method.

**Figure S10.** Comparison of chromatograms obtained from untargeted LC-MS analysis using different gradient curve parameter values for method optimisation. Each colour represents a solvent used for extraction with signal intensities shown. The colours are represented by the following solvents: black – gradient curve of 3 (2.17E9), red – 5 (1.96E9), green – 7 (1.84E9).

**Figure S11.** The impact of gradient curve on untargeted urinary metabolomics. Outcomes were assessed by **(A)** chromatographic visualisation, **(B)** the number of metabolites detected, **(C)** their average peak rating, **(D)** the area under the curve (AUC) of the detected creatinine peak and **(E)** the associated peak rating. The peak performance of creatinine was further evaluated, as measured by the quantification of peak quality factors (PQF)s, **(F)** FWHM2Base, **(G)** Jaggedness, and **(H)** Modality indices. The chromatogram presented in (A) represents the optimised method parameter selected for the method. The zig-zag indices are not shown as all parameters tested gave a zero value.

**Table S4**. Untargeted metabolomics experiment elution gradient. Mobile phase A, 99.9% water + 0.1% formic acid, mobile phase B, 99.9% ACN + 0.1% formic acid.

| Time (min) | Mobile Phase A (%) | Mobile Phase B (%) | Flow rate (mL/min) | Gradient curve |
| --- | --- | --- | --- | --- |
| 0.0 | 99.0 | 1.0 | 0.4 | 7 |
| 0.5 | 99.0 | 1.0 | 0.4 | 7 |
| 4.0 | 50.0 | 50.0 | 0.4 | 7 |
| 6.0 | 5.0 | 55.0 | 0.4 | 7 |
| 6.01 | 1.0 | 99.0 | 0.4 | 7 |
| 8.0 | 1.0 | 99.0 | 0.4 | 7 |
| 9.5 | 99.0 | 1.0 | 0.4 | 7 |
| 10.0 | 99.0 | 1.0 | 0.4 | 7 |

**Figure S12.** Comparison of full scan AGC parameters and their impact on untargeted urinary metabolomics. Outcomes were assessed by **(A)** the number of metabolites detected, **(B)** their average peak rating, **(C)** the area under the curve (AUC) of the detected creatinine peak and **(D)** the associated peak rating. The peak performance of creatinine was further evaluated, as measured by the quantification of peak quality factors (PQF)s, **(E)** FWHM2Base, **(F)** Jaggedness, and **(G)** Modality indices. The zig-zag indices are not shown as all parameters tested gave a zero value.

**Figure S13.** Comparison of MSMS scan AGC parameters and their impact on untargeted urinary metabolomics. Outcomes were assessed by **(A)** the number of metabolites detected, **(B)** their average peak rating, **(C)** the area under the curve (AUC) of the detected creatinine peak and **(D)** the associated peak rating. The peak performance of creatinine was further evaluated, as measured by the quantification of peak quality factors (PQF)s, **(E)** Jaggedness, **(F)** Modality, and **(G)** FWHM2Base indices. The zig-zag indices are not shown as all parameters tested gave a zero value.

**Figure S14**. Final optimised workflow for the analysis of urine samples using untargeted UHPLC-MS and application of the optimised method to a clinical trial for urine metabolomics analysis. Schematic representation of the optimised method, which includes the selection of specific sample preparation and LC-MS parameters to ensure the comprehensive and reproducible profiling of urinary metabolites. Illustration of the clinical trial workflow, consisting of three independent studies which aimed to measure the global urine metabolic profile. The total analysis time of the urine samples for all three studies was 182.3 hours and over 1500 metabolites were putatively identified.

B

A

C

**Figure S15**. Principal component analysis (PCA) plots comparing three normalisation strategies, evaluating two study groups. (A) Non-normalised, (B) Creatinine normalised, and (C) PQN normalised urine data.

**Table S5**. Coefficient of Variation (CV) values for metabolites detected in three normalisation methods. Metabolites were detected at MSI level 2.

| Metabolite | Coefficient of Variation (CV) | | |
| --- | --- | --- | --- |
|  | Non-Normalised | Creatinine Normalised | PQN Normalised |
| Acadesine | 0.331688 | 0.560431 | 0.370701 |
| Acetaminophen glucuronide | 0.164351 | 0.223531 | 0.104566 |
| Acetanilide | 0.248759 | 1.711 | 2.520153 |
| Acetoacetic acid | 0.155875 | 0.803121 | 2.009837 |
| Acetyl-L-carnitine | 0.342468 | 0.300348 | 0.298355 |
| Acetylagmatine | 0.391684 | 0.587913 | 0.416512 |
| Acronycidine | 0.2768 | 0.26706 | 0.250245 |
| Adenosine | 1.25527 | 1.349067 | 1.266701 |
| Aderbasib | 0.424737 | 0.510881 | 0.40471 |
| Ala-pro | 0.31781 | 0.295325 | 0.268673 |
| alangiside | 0.256917 | 0.296218 | 0.246416 |
| Alanyltyrosine | 0.340473 | 0.280605 | 0.292433 |
| alloxydim | 0.327958 | 0.367294 | 0.301518 |
| Allyl cyclohexanevalerate | 0.211445 | 0.293483 | 0.278732 |
| Allyl undecylenate | 0.185418 | 0.153912 | 0.110492 |
| alpha-CEHC | 0.150504 | 0.52575 | 1.339858 |
| Aminoadipic acid | 0.497858 | 0.423307 | 0.491964 |
| Amoxicillin | 0.150426 | 0.285977 | 0.13401 |
| Amylbenzene | 0.190241 | 0.198508 | 0.124037 |
| Anhydrovitamin A | 0.192672 | 0.196269 | 0.19329 |
| Ankorine | 0.318115 | 0.360032 | 0.305704 |
| anticapsin | 0.220638 | 0.140762 | 0.136709 |
| Apiin | 0.184699 | 0.182665 | 0.103117 |
| Aprobarbital | 0.714812 | 2.368973 | 3.805627 |
| Arginyltyrosine | 0.992742 | 1.043739 | 1.058323 |
| Artesunate | 0.154425 | 0.206209 | 0.088057 |
| Ascorbic acid | 0.676673 | 0.651875 | 0.631369 |
| asp-gln | 0.420086 | 0.35792 | 0.367315 |
| asp-leu | 0.206243 | 0.387374 | 0.227824 |
| Asp-tyr | 0.380151 | 0.315801 | 0.331717 |
| Aspartame | 0.147943 | 0.199428 | 0.107807 |
| Aspartyl-L-proline | 0.253005 | 0.336906 | 0.230723 |
| Aspartylphenylalanine | 0.144494 | 0.198865 | 0.102862 |
| Asymmetric dimethylarginine | 1.375601 | 1.462349 | 1.701033 |
| Azathioprine | 0.141684 | 0.271926 | 0.120996 |
| Bakankosin | 0.183495 | 0.220842 | 0.120613 |
| Benzaldehyde | 0.159799 | 0.17218 | 0.329652 |
| Benzenepentol | 0.223226 | 0.23625 | 0.190758 |
| Benzyl 6-O-D-xylopyranosyl-D-glucopyranoside | 0.186646 | 0.232944 | 0.128437 |
| Bicyclomycin | 0.47775 | 0.420024 | 0.423532 |
| Bilirubin | 0.270926 | 0.339281 | 0.384622 |
| Binapacryl | 0.393696 | 0.456469 | 0.37949 |
| Biopterin | 0.495612 | 0.653828 | 0.512979 |
| Biotin | 0.209777 | 0.151497 | 0.14408 |
| Biotin sulfone | 0.559814 | 0.57346 | 0.542461 |
| bispyribac | 0.870502 | 0.922125 | 0.868396 |
| Boschnaloside | 0.149748 | 0.20617 | 0.090828 |
| Buchananine | 0.53707 | 0.431021 | 0.477016 |
| Bufadienolide | 0.280447 | 0.345598 | 0.270061 |
| Buflomedil | 0.338015 | 0.379196 | 0.326198 |
| Butenylcarnitine | 0.283405 | 0.194321 | 0.225307 |
| butilfenin | 0.157854 | 0.19916 | 0.072822 |
| butoctamide | 0.609991 | 0.689398 | 0.642857 |
| Butyl 3-(2-butoxy-2-oxoethyl)-3-hydroxy-4-oxo-2-oxetanecarboxylate | 0.191025 | 0.223066 | 0.16371 |
| C12-Carnitine | 0.610126 | 0.603067 | 0.578048 |
| Capric acid | 0.286486 | 0.310978 | 0.38058 |
| Caprolactam | 0.18946 | 1.590292 | 2.806307 |
| Capryloylglycine | 0.162916 | 0.816068 | 1.555328 |
| carbapenem MM22383 | 0.166343 | 0.189836 | 0.112196 |
| carbazeran | 0.150181 | 0.202463 | 0.083905 |
| Carbidopa | 0.154507 | 0.274143 | 0.126903 |
| Carbinoxamine | 0.481381 | 0.458593 | 0.674575 |
| Carboxy-ibuprofen | 0.766323 | 0.882086 | 0.778611 |
| Carboxytolbutamide | 0.189998 | 0.322531 | 0.184449 |
| Carminomycin I | 0.288822 | 0.490485 | 0.840031 |
| Carvone | 0.275154 | 0.34754 | 0.27638 |
| Cassaidine | 0.170115 | 0.171355 | 0.164251 |
| Cervonoyl ethanolamide | 0.149239 | 0.208235 | 0.175742 |
| Cetirizine | 0.175663 | 0.184145 | 0.12115 |
| Cetraxate | 0.259837 | 0.248949 | 0.191821 |
| Chlorpheniramine | 0.435135 | 0.337113 | 0.433725 |
| Ciclopirox | 0.480207 | 1.704539 | 3.007247 |
| Cinnamoylglycine | 0.149903 | 0.195858 | 0.118189 |
| cis-trihomoaconitic acid | 0.204978 | 0.304273 | 0.159975 |
| Citric acid | 0.468642 | 0.703948 | 0.509385 |
| Coronatine | 0.29638 | 0.340257 | 0.424193 |
| Corticosterone | 0.429358 | 0.432366 | 0.409621 |
| Cortisol | 0.178864 | 0.236692 | 0.131256 |
| Creatine | 0.203362 | 0.104461 | 0.142706 |
| Creatinine | 0.205989 | 0.205989 | 0.408276 |
| crinamidine | 0.350479 | 0.360186 | 0.3418 |
| Crotonic acid | 1.772563 | 1.740595 | 1.810214 |
| Cyclamic acid | 0.366809 | 0.291482 | 0.317842 |
| cyclic Melatonin | 0.181304 | 0.178561 | 0.127048 |
| Cyclo(leucylleucine) | 0.339559 | 0.437208 | 0.317552 |
| Cyclohexylamine | 0.335535 | 1.149178 | 2.412831 |
| cypendazole | 0.47187 | 0.40928 | 0.470699 |
| Cyprodenate | 0.158089 | 0.202698 | 0.100317 |
| cys-his | 0.210014 | 0.224411 | 0.162586 |
| Cytarabine | 0.256925 | 0.348519 | 0.228832 |
| D-(+)-Pyroglutamic Acid | 0.235877 | 0.516011 | 1.867827 |
| D-(+)-Tryptophan | 0.155632 | 0.301537 | 0.159441 |
| D-Lysyl-L-valyl-D-valyl-L-allothreonine | 0.201977 | 1.356479 | 2.08465 |
| Decanohydroxamic acid | 0.14702 | 0.212356 | 0.168328 |
| Decanoylcarnitine | 0.305246 | 0.327253 | 0.280795 |
| delcorine | 0.309998 | 0.344542 | 0.361682 |
| Demethylalangiside | 0.217644 | 0.231229 | 0.149617 |
| deoxyhypusine | 0.321101 | 0.230469 | 0.267052 |
| Desloratadine | 0.169599 | 1.285405 | 2.294232 |
| Dhurrin | 0.843351 | 0.777095 | 0.847057 |
| Diaminopimelic acid | 0.508944 | 0.425101 | 0.466074 |
| diethyl oxalpropionate | 0.159229 | 0.219939 | 0.077385 |
| Diethylcarbamazine N-oxide | 0.249947 | 0.221475 | 0.218731 |
| dihomomethionine | 0.201114 | 0.20775 | 0.151185 |
| Dihydrocaffeic acid 3-O-glucuronide | 0.261502 | 0.407591 | 0.605558 |
| Dihydroconiferin | 0.165359 | 0.219242 | 0.097977 |
| Dihydrothymine | 0.204331 | 0.066222 | 0.249492 |
| Dihyroxy-1H-indole glucuronide I | 0.388787 | 0.392393 | 0.344731 |
| Dinoseb | 0.353421 | 0.404443 | 0.316515 |
| Dipivefrin | 0.210077 | 0.26613 | 0.166644 |
| DL-Carnitine | 0.244206 | 0.102423 | 0.184625 |
| DL-Aminocaprylic acid | 0.167564 | 0.191454 | 0.11783 |
| Docosahexaenoic acid ethyl ester | 0.211067 | 0.23706 | 0.176818 |
| Dodecanedioic acid | 0.148114 | 0.181343 | 0.065507 |
| Dopamine | 2.489068 | 2.848741 | 3.345976 |
| Dynone | 0.490969 | 0.415169 | 0.475163 |
| Ecgonine | 0.154566 | 0.213666 | 0.353273 |
| Ecgonine methyl ester | 0.175907 | 0.362642 | 0.799798 |
| Ectoine | 0.318361 | 0.213948 | 0.261204 |
| Elacytarabine | 0.489785 | 0.473824 | 0.588573 |
| Epinephrine | 0.147356 | 0.177008 | 0.06129 |
| Epinephrine glucuronide | 1.886223 | 2.002812 | 1.924165 |
| Epinephrine sulfate | 0.413029 | 0.439102 | 0.394113 |
| Epithienamycin E | 0.849786 | 0.744306 | 0.804779 |
| Ergonovine | 0.213346 | 0.179154 | 0.15519 |
| Esmolol | 0.246455 | 0.217002 | 0.193084 |
| Eterobarb | 0.161949 | 0.203795 | 0.09059 |
| Ethychlozate | 0.166104 | 0.214088 | 0.252722 |
| Ethyl {2-[(2E)-5-(hexopyranosyloxy)-2-penten-1-yl]-3-oxocyclopentyl}acetate | 0.276538 | 0.343872 | 0.489263 |
| Ethyl butylacetylaminopropionate | 0.157822 | 0.222262 | 0.248788 |
| Ethyl methylphenylglycidate | 0.419673 | 0.538436 | 0.422145 |
| Ethylbenzene | 0.160197 | 0.204977 | 0.09234 |
| Ethylenediamine-N,N'-diacetic acid | 0.297495 | 0.294168 | 0.259281 |
| Fenethylline | 0.156587 | 0.192454 | 0.082502 |
| Fenitropan | 0.158359 | 0.193679 | 0.072068 |
| Ferulic acid | 0.184779 | 0.218278 | 0.488299 |
| Fesoterodine | 0.187629 | 0.163314 | 0.112165 |
| Fexofenadine | 0.159324 | 0.197178 | 0.147096 |
| fosfocreatinine | 0.244949 | 0.174223 | 0.207969 |
| Fraxin | 0.18396 | 0.282202 | 0.406463 |
| Furmecyclox | 0.285387 | 0.329559 | 0.266969 |
| Fusarochromanone | 0.203979 | 0.179944 | 0.108891 |
| Gabexate | 0.193036 | 0.182213 | 0.174989 |
| Galantamine | 0.241581 | 0.162279 | 0.229246 |
| Gentisuric acid | 0.483957 | 0.41269 | 0.439795 |
| gibberellin A(3) O-D-glucoside | 0.264389 | 0.391938 | 0.287664 |
| glaucarubinone | 0.279087 | 0.270198 | 0.254165 |
| Gln-Gln | 0.537328 | 0.492761 | 0.510629 |
| gln-phe | 0.276119 | 0.264919 | 0.443854 |
| Glucosylgalactosyl hydroxylysine | 0.337886 | 0.247756 | 0.332777 |
| Glutarylcarnitine | 0.345741 | 0.507643 | 0.386649 |
| Gly-DL-Phe | 0.255917 | 0.444448 | 0.278562 |
| Glycinexylidide | 0.20543 | 0.269173 | 0.16598 |
| Glycodiazine | 0.183288 | 0.278163 | 0.152087 |
| Glycyl-L-asparaginyl-D-leucine | 0.182829 | 0.193662 | 0.117845 |
| Glycylglutamine | 0.433104 | 0.345923 | 0.388222 |
| Glycylproline | 0.296133 | 0.237142 | 0.243368 |
| Guanadrel | 0.238543 | 0.309154 | 0.194018 |
| Guvacoline | 0.177359 | 0.364491 | 2.119097 |
| Gynocardin | 0.460024 | 0.660554 | 0.483235 |
| Hawkinsin | 0.627212 | 0.536181 | 0.602509 |
| HC Blue 1 | 0.162341 | 0.246389 | 0.127131 |
| HC blue 2 | 0.487953 | 0.441689 | 0.45446 |
| Heliotron | 0.729528 | 0.688243 | 0.7117 |
| Heptylbenzene | 0.280753 | 0.23201 | 0.266212 |
| hercynylcysteine sulfoxide | 0.296864 | 0.307135 | 0.276555 |
| Hesperetin 7-O-glucuronide | 0.290197 | 0.248552 | 0.228185 |
| Hexadecanedioic acid mono-L-carnitine ester | 0.739949 | 0.643359 | 0.677168 |
| Hexadienic acid | 0.275793 | 1.459118 | 2.639353 |
| hexahomomethionine | 0.287371 | 0.31689 | 0.243606 |
| Hexanoylcarnitine | 0.298564 | 0.34525 | 0.276389 |
| Hippuric acid | 0.142487 | 0.204384 | 0.077657 |
| His-pro | 0.373188 | 0.419367 | 0.449158 |
| Histamine | 0.289122 | 0.191359 | 0.229764 |
| Histidinol | 0.247367 | 0.384297 | 0.675695 |
| Homo-L-arginine | 0.1958 | 0.136096 | 0.173443 |
| Hostmaniane | 0.161938 | 0.446571 | 0.636659 |
| Hydroxyphenylacetylglycine | 0.28416 | 0.314002 | 0.27093 |
| Hymexazol N-glucoside | 0.532662 | 0.595535 | 0.516619 |
| Hymexazol O-glucoside | 0.380657 | 0.323573 | 0.338524 |
| Hypusine | 0.268522 | 0.182997 | 0.198424 |
| Imidazolelactic acid | 0.218811 | 0.091507 | 0.296764 |
| Indicine | 0.472134 | 0.432033 | 0.535858 |
| indol-2-one | 0.167712 | 0.300046 | 0.140461 |
| Indole-3-acetic-acid-O-glucuronide | 0.147009 | 0.205831 | 0.104993 |
| Indole-3-lactic acid | 0.154059 | 0.20254 | 0.111446 |
| Indoleacetyl glutamic acid | 0.163304 | 0.196731 | 0.100034 |
| Indoleacetyl glutamine | 0.14718 | 0.200903 | 0.105389 |
| Indoleacetylaspartate | 0.165199 | 0.177339 | 0.056579 |
| Indoxyl-D-glucuronide | 0.171099 | 0.275101 | 0.131407 |
| Ingenol mebutate | 0.228816 | 0.319057 | 0.22163 |
| Integerrimine | 0.159928 | 0.184717 | 0.107912 |
| Isoamylamine | 0.45038 | 1.967554 | 3.277011 |
| Isocromadurine | 0.18373 | 0.175259 | 0.104885 |
| Isoetharine | 0.194218 | 0.266554 | 0.155272 |
| Isofraxidin | 0.229667 | 0.290378 | 0.445235 |
| Isohomovanillic acid | 0.189483 | 0.205123 | 0.132522 |
| Isopropyl D-galactopyranoside | 0.281203 | 0.622895 | 1.212605 |
| Istamycin C1 | 0.159864 | 0.590179 | 1.041499 |
| Jasmonic acid | 0.826192 | 2.410488 | 3.711872 |
| L-(-)-Methionine | 0.193416 | 0.253963 | 0.229807 |
| L-(+)-Citrulline | 0.290849 | 0.193134 | 0.237147 |
| L-dihydroanticapsin | 0.76921 | 0.714401 | 0.738968 |
| L-Dopa | 0.635251 | 0.54845 | 0.597785 |
| L-Fucose | 0.394307 | 0.32819 | 0.335018 |
| L-Glutamine | 0.16719 | 0.28502 | 0.160159 |
| L-Hexanoylcarnitine | 0.179523 | 0.214972 | 0.115176 |
| L-Histidine | 0.299082 | 0.357517 | 0.720725 |
| L-Kynurenine | 0.241936 | 0.173924 | 0.279605 |
| L-Lysine | 0.253939 | 0.259965 | 0.692792 |
| L-Norleucine | 0.523615 | 1.325822 | 1.933269 |
| L-Phenylalanine | 0.223008 | 0.138711 | 0.165768 |
| L-Prolyl-4-hydroxy-L-prolin | 0.486566 | 0.400441 | 0.439451 |
| L-Tyrosine | 0.67572 | 0.649253 | 0.653036 |
| L-Tyrosyl-L-prolyl-L-tryptophyl-L-threonine | 0.27627 | 0.293397 | 0.202258 |
| L-Valine | 0.251274 | 2.326997 | 3.576211 |
| L-Glutamyl-L-valine | 0.242976 | 0.150224 | 0.191378 |
| Lacosamide | 0.362168 | 0.358232 | 0.329935 |
| Lenticin | 0.16022 | 0.209501 | 0.108546 |
| leu-gln | 0.942871 | 0.887265 | 0.91859 |
| Leu-Val | 0.378883 | 0.296812 | 0.339353 |
| Leucylasparagine | 0.423208 | 0.352995 | 0.407638 |
| Leucylphenylalanine | 0.163508 | 0.209994 | 0.07569 |
| Leucylproline | 0.148412 | 0.235863 | 0.094682 |
| Levetiracetam | 2.381772 | 2.229663 | 2.246061 |
| Levodropropizine | 0.322456 | 0.361108 | 0.372482 |
| Lidocaine | 0.138593 | 0.208654 | 0.109192 |
| Lincomycin | 0.18303 | 0.245908 | 0.113663 |
| Lorbamate | 0.151322 | 0.197298 | 0.10497 |
| Lovastatin | 0.151619 | 0.183767 | 0.10349 |
| lys-leu | 0.396623 | 0.492484 | 0.394544 |
| Lysylvaline | 0.186148 | 0.280986 | 0.166515 |
| Maculosin | 0.165839 | 0.166447 | 0.091521 |
| Malonylcarnitine | 0.412071 | 0.328398 | 0.370772 |
| Mercaptopurine | 0.319764 | 0.226919 | 0.257342 |
| merimepodib | 0.4767 | 0.511171 | 0.461507 |
| Metanephrine | 0.159321 | 0.184378 | 0.083612 |
| Metaproterenol | 0.202729 | 0.188581 | 0.139127 |
| Metharbital | 0.304391 | 0.178655 | 0.246544 |
| Methotrexate | 0.171295 | 0.226753 | 0.11013 |
| Methyl (3aR,5Z,9E,11aS)-10-methyl-3-methylene-2-oxo-2,3,3a,4,7,8,11,11a-octahydrocyclodeca[b]furan-6-carboxylate | 0.15818 | 0.246242 | 0.263893 |
| Methyl {2-[(2E)-5-(hexopyranosyloxy)-2-penten-1-yl]-3-oxocyclopentyl}acetate | 0.150116 | 0.221298 | 0.082561 |
| methyl 1-methyl-1,2,5,6-tetrahydropyridine-3-carboxylate hydrobromide | 0.428445 | 0.364538 | 0.542968 |
| methyl 2-(benzoylamino)acetate | 0.151156 | 0.201633 | 0.070434 |
| Methyl 2-deoxy-3-O-(4-deoxy-4-methyl-D-glucopyranuronosyl)-2-[(Z)-(1-hydroxyethylidene)amino]-D-galactopyranoside | 0.539998 | 0.610709 | 0.673654 |
| Methyl 2,3-dihydro-3-hydroxy-2-oxo-1H-indole-3-acetate | 0.286039 | 0.471623 | 0.853315 |
| Methyl O-sulfo-L-tyrosinate | 0.36435 | 0.535876 | 0.412776 |
| Methylimidazoleacetic acid | 0.206398 | 0.254213 | 1.507307 |
| Methylone | 0.16921 | 0.161331 | 0.071626 |
| Methylphenidate | 0.154032 | 0.421352 | 0.617855 |
| Metronidazole | 0.184129 | 0.330791 | 0.187088 |
| Miglustat | 0.227881 | 0.369665 | 0.224221 |
| Miraxanthin-II | 0.219989 | 0.197496 | 0.222078 |
| Monobutyl phthalate | 0.160886 | 0.225703 | 0.132681 |
| Monocrotaline | 0.185007 | 0.230135 | 0.114397 |
| Mycalamide A | 0.148155 | 0.186483 | 0.099777 |
| Myriocin | 0.151595 | 0.195201 | 0.113412 |
| Myxalamid A | 0.252481 | 0.284929 | 0.208852 |
| Myxochelin A | 0.514659 | 0.57637 | 0.83752 |
| N-(2-Amino-3-phenylpropanoyl)glutamine | 0.145898 | 0.190044 | 0.077515 |
| N-(2-Furylmethyl)-7-(D-glucopyranosyl)-7H-purin-6-amine | 1.248388 | 1.390662 | 1.258077 |
| N-(2,3,4-Trimethoxybenzoyl)glycine | 0.545433 | 0.451145 | 0.536334 |
| N-(3-Carboxypropanoyl)-5-hydroxynorvaline | 0.204847 | 0.305035 | 0.159285 |
| N-(4-Heptanyl)-1,3-benzodioxole-5-carboxamide | 0.151245 | 0.171053 | 0.093215 |
| N-(N-(3-Amino-3-carboxypropyl)-3-amino-3-carboxypropyl)azetidine-2-carboxylic acid | 0.156409 | 0.274021 | 0.146954 |
| N-[(1S)-4-Carbamimidamido-1-carboxybutyl]asparaginylaspartic acid | 0.199077 | 0.286247 | 0.152527 |
| N-[(5S)-5-Amino-5-carboxypentanoyl]cysteinyl-D-valine | 0.243922 | 0.248057 | 0.179768 |
| N-[3-Carboxy-2-(carboxymethyl)-2-hydroxypropanoyl]glutamic acid | 0.67086 | 0.879566 | 0.722492 |
| N-[4-(5-Amino-2,2-dimethyl-4-oxo-3,4-dihydro-2H-chromen-6-yl)-1-hydroxy-4-oxo-2-butanyl]acetamide | 0.484051 | 0.710285 | 1.165616 |
| N-[4'-hydroxy-(E)-cinnamoyl]-L-aspartic acid | 0.192549 | 0.297121 | 0.290482 |
| N-{3-[(4-Acetamidobutyl)amino]propyl}acetamide | 0.363407 | 0.590073 | 0.399255 |
| N-a-Acetyl-L-arginine | 0.281418 | 0.200804 | 0.22753 |
| N-Acetyl-1-aspartylglutamic acid | 0.27831 | 0.387451 | 0.276376 |
| N-Acetyl-D-lactosamine | 0.252648 | 0.255438 | 0.321597 |
| N-Acetyl-L-aspartic acid | 0.438187 | 0.564806 | 0.444441 |
| N-Acetyl-L-carnosine | 0.337344 | 0.265729 | 0.28774 |
| N-Acetyl-L-cysteine | 0.284255 | 0.302959 | 0.263248 |
| N-Acetyl-L-glutamic acid | 0.283404 | 0.431851 | 0.298402 |
| N-Acetyl-L-histidine | 0.263607 | 0.167046 | 0.635833 |
| N-Acetyl-L-leucine | 0.148272 | 0.201481 | 0.192337 |
| N-Acetyl-L-phenylalanine | 0.222097 | 0.214958 | 0.158961 |
| N-Acetyl-L-tyrosine | 0.146561 | 0.226592 | 0.102458 |
| N-acetyl-S-(N-allylthiocarbamoyl)-L-cysteine | 0.157649 | 0.192294 | 0.090869 |
| N-Acetyl-S-2-hydroxyethyl-L-cysteine | 0.355982 | 0.321149 | 0.315127 |
| N-Acetyl-D-glucosamine | 0.521256 | 0.541227 | 0.96582 |
| N-Acetylglutamine | 0.399395 | 0.375086 | 0.364154 |
| N-Acetylhistamine | 0.277219 | 0.173322 | 0.21545 |
| N-Acetylleucylleucine | 0.230877 | 0.165707 | 0.153911 |
| N-Acetylneuraminic acid | 0.52682 | 3.481264 | 4.566382 |
| N-Acetylputrescine | 0.25504 | 0.147585 | 0.339673 |
| N-Acetylserotonin | 0.167842 | 0.29944 | 0.138583 |
| N-Acetyltryptophan | 0.140351 | 0.176916 | 0.084884 |
| N-Acetylvanilalanine | 0.191411 | 0.223194 | 0.145415 |
| N-Formyl-L-methionine | 0.368457 | 0.372596 | 0.327426 |
| N-lauroylglycine | 0.189277 | 0.23854 | 0.150151 |
| N-Methylhydantoin | 0.513828 | 2.040917 | 3.34829 |
| N-Methyltryptamine | 0.167143 | 0.285684 | 0.140871 |
| N-Nitrosodibutylamine | 0.227034 | 0.248912 | 0.198799 |
| N-Phenyl-D-glucopyranosylamine | 0.204544 | 0.180672 | 0.141962 |
| N-Phenylacetylglutamic acid | 0.149707 | 0.190315 | 0.103635 |
| N-Phenylacetylglutamine | 0.14445 | 0.189237 | 0.109358 |
| N-Phenylacetylphenylalanine | 0.15486 | 0.168287 | 0.0908 |
| N-Propionylmethionine | 0.175014 | 0.180023 | 0.104914 |
| N-Undecanoylglycine | 0.233495 | 2.57094 | 4.182982 |
| N,N-Bis(2-hydroxyethyl)dodecanamide | 1.089717 | 4.574854 | 6.352043 |
| N~2~-(Carboxymethyl)arginine | 0.296249 | 0.206247 | 0.252059 |
| N~6~-Octanoyllysine | 0.184044 | 0.204302 | 0.127928 |
| N2-Acetylornithine | 0.37063 | 0.259675 | 0.317642 |
| N2-Methylguanosine | 0.647697 | 0.629113 | 0.635784 |
| N4-Acetylcytidine | 0.174486 | 0.184393 | 0.122642 |
| N6-Acetyl-L-lysine | 0.323827 | 0.215362 | 0.280305 |
| N6-Me-Adenosine | 0.226859 | 0.234606 | 0.1859 |
| N6-threonylcarbamoyladenosine | 0.152505 | 0.202709 | 0.116409 |
| N8-Acetylspermidine | 0.340977 | 0.210551 | 0.27856 |
| Nadolol | 0.20111 | 0.238502 | 0.216754 |
| Nalidixic acid | 0.176426 | 0.193687 | 0.132602 |
| NAPQI | 0.164552 | 0.23256 | 0.10309 |
| Naringenin 5-O-glucuronide | 0.169561 | 0.202194 | 0.151012 |
| Naringeninchalcone | 0.236606 | 0.201699 | 0.281431 |
| Neopterin | 0.451713 | 0.408871 | 0.410897 |
| Nicotine | 0.283076 | 0.246242 | 0.34983 |
| Nicotinic acid | 0.20623 | 0.416905 | 0.860414 |
| Nicotinuric acid | 0.139317 | 0.233614 | 0.082143 |
| Nigakilactone N | 0.392138 | 0.481155 | 0.424544 |
| Nikethamide | 0.201938 | 0.252871 | 0.192334 |
| Nipradilol | 0.22576 | 0.225399 | 0.165233 |
| Nitrendipine | 0.177238 | 0.186977 | 0.099717 |
| Nitrosoguvacoline | 0.24415 | 0.143701 | 0.193018 |
| Nivalenol | 0.172226 | 0.222967 | 0.10488 |
| Nonanoylcarnitine | 0.233895 | 5.075334 | 6.13448 |
| Norepinephrine sulfate | 0.295327 | 0.471569 | 0.313336 |
| Norlidocaine | 0.229815 | 0.32373 | 0.210802 |
| NP-001346 | 0.158958 | 0.229062 | 0.124838 |
| NP-008998 | 0.354106 | 1.386893 | 2.041588 |
| NP-015114 | 0.227729 | 0.343428 | 0.218186 |
| NP-022229 | 0.331123 | 0.35676 | 0.302823 |
| nylon cyclic dimer | 0.314564 | 0.214933 | 0.276207 |
| O-(4,8-dimethylnonanoyl)carnitine | 0.410737 | 0.461419 | 0.399308 |
| O-heptanoylcarnitine | 0.144191 | 0.192052 | 0.117882 |
| O-ureido-D-serine | 0.330518 | 0.252138 | 0.448852 |
| Ondansetron | 0.179822 | 0.243813 | 0.127262 |
| ophthalmic acid | 0.238684 | 0.150508 | 0.183106 |
| ornithinoalanine | 0.242802 | 0.309088 | 0.199134 |
| Oxamniquine | 0.266799 | 0.343666 | 0.235679 |
| Oxepanone | 0.202331 | 0.329517 | 0.284829 |
| Oxirane, 2-(6-heptenyl)-3-(2,4-pentadiynyl)- | 0.963779 | 0.957275 | 0.944684 |
| Oxprenolol | 0.158189 | 0.264899 | 0.127755 |
| p-Cresol | 0.164323 | 0.238606 | 0.413346 |
| Panthenol | 0.182662 | 0.25095 | 0.149079 |
| Pantothenic acid | 0.194529 | 0.354304 | 0.195236 |
| Paracetamol | 0.157065 | 0.287579 | 0.254364 |
| Paracetamol-cysteine | 0.196659 | 0.152786 | 0.144436 |
| Paucin | 0.198271 | 0.306611 | 0.198089 |
| pC-HSL | 0.270358 | 0.386706 | 0.273914 |
| PEG n6 | 0.110793 | 1.136993 | 2.076918 |
| PEG n7 | 0.163204 | 0.538931 | 0.974217 |
| PEG n8 | 0.165874 | 0.482854 | 0.82985 |
| Peimine | 0.149702 | 0.215384 | 0.104081 |
| Pentadecanoylcarnitine | 0.193626 | 0.229951 | 0.212569 |
| pentahomomethionine | 0.1745 | 0.2415 | 0.142768 |
| pentigetide | 0.23274 | 0.213495 | 0.169501 |
| Perindopril | 0.186217 | 0.248316 | 0.139696 |
| Phendimetrazine | 0.177205 | 0.297735 | 0.387348 |
| Phenol | 0.145026 | 0.192206 | 0.096181 |
| Phenylacetylglutamine | 0.652851 | 0.707634 | 0.996101 |
| Phenylglucuronide | 0.178633 | 0.299124 | 0.154602 |
| Pilocarpine | 0.171152 | 0.249467 | 0.113951 |
| Pindolol | 0.223284 | 0.189929 | 0.164897 |
| Pipecolic acid | 0.560622 | 0.715232 | 1.240568 |
| Pirfenidone | 0.304433 | 0.40387 | 0.770898 |
| Prednisolone | 0.172187 | 0.245322 | 0.136126 |
| pretyrosine | 0.351671 | 0.446817 | 0.341285 |
| Prilocaine | 0.141257 | 0.22835 | 0.107283 |
| Primidolol | 0.140712 | 0.219276 | 0.068338 |
| Proacaciberin | 0.206209 | 0.235968 | 0.148355 |
| prohydrojasmon | 0.180909 | 0.214508 | 0.131466 |
| Proline | 0.335728 | 0.328209 | 0.54103 |
| promolate | 0.232785 | 0.249983 | 0.172716 |
| Proparacaine | 0.23695 | 0.244662 | 0.242576 |
| Propionylcarnitine | 0.311119 | 0.199369 | 0.263527 |
| Pulcherriminic acid | 0.151327 | 0.201255 | 0.106939 |
| Pyridoxal | 0.214617 | 0.19916 | 0.262255 |
| Pyridoxamine | 0.375743 | 0.853103 | 1.510623 |
| Pyridoxine | 0.359944 | 1.206134 | 2.313478 |
| Pyrogallol-2-O-glucuronide | 0.629119 | 0.612531 | 0.609958 |
| Pyrraline | 0.187039 | 0.352668 | 0.19961 |
| Quinoline | 0.147601 | 0.203286 | 0.102019 |
| Rehmaionoside C | 0.143138 | 0.220702 | 0.132793 |
| Reproterol | 0.562148 | 0.685498 | 0.640339 |
| Resorcinol diglycidyl ether | 0.187084 | 0.18282 | 0.10774 |
| Retinoyl b-glucuronide | 0.363992 | 0.473172 | 0.390087 |
| Riboflavin reduced | 0.170393 | 0.218638 | 0.230438 |
| Ricinine | 0.206369 | 0.511904 | 1.034023 |
| Riddelliine | 0.161288 | 0.211871 | 0.105205 |
| Rimexolone | 0.167803 | 0.184154 | 0.178869 |
| Ritalinic acid | 0.14142 | 0.204418 | 0.096882 |
| Rosmarinine | 0.200348 | 0.214554 | 0.128489 |
| S-(Hydroxyphenylacetothiohydroximoyl)-L-cysteine | 0.408751 | 0.453504 | 0.420195 |
| S-{[(1aR,7aS,10aS,10bR)-1a,5-Dimethyl-9-oxo-1a,2,3,6,7,7a,8,9,10a,10b-decahydrooxireno[9,10]cyclodeca[1,2-b]furan-8-yl]methyl}-L-cysteine | 0.465386 | 0.485365 | 0.482862 |
| S-3-oxodecanoyl cysteamine | 0.338514 | 0.390078 | 0.32874 |
| S-Adenosylhomocysteine | 0.602834 | 0.831539 | 0.643527 |
| S-Allylcysteine | 0.162633 | 0.188669 | 0.103062 |
| Salbutamol | 0.190686 | 0.212488 | 0.125309 |
| Salbutamol 4-O-sulfate | 0.517906 | 0.468755 | 0.56292 |
| salicyluric D-glucuronide | 0.219252 | 0.29954 | 0.172677 |
| Salidroside | 0.502534 | 0.530941 | 0.549261 |
| salinosporamide B | 0.163227 | 0.160661 | 0.09026 |
| Salsolinol | 0.194174 | 0.189226 | 0.213657 |
| Sebacic acid | 0.156706 | 0.220601 | 0.080734 |
| Sedanolide | 0.139127 | 0.820194 | 1.358763 |
| Senkyunolide | 0.85224 | 1.293216 | 2.577889 |
| Sertraline | 0.341986 | 1.318373 | 2.418345 |
| Sinapinic acid | 0.175468 | 0.254403 | 0.117383 |
| sinapoyltartronic acid | 0.314356 | 0.215867 | 0.272873 |
| Solasodine | 0.150339 | 0.200107 | 0.087041 |
| Sorbitan, monododecanoate | 0.210312 | 0.236926 | 0.198781 |
| Spermidine | 0.400294 | 0.340448 | 0.361949 |
| Spermine | 0.511136 | 0.484167 | 0.572616 |
| streptobiosamine | 0.215078 | 0.257041 | 0.164968 |
| Suberic acid | 0.14676 | 0.744518 | 3.657796 |
| Suberylglycine | 0.144507 | 0.210205 | 0.098062 |
| Succinyladenosine | 0.182323 | 0.352256 | 0.194563 |
| Succinylcarnitine | 0.197236 | 0.194728 | 0.114244 |
| Sulcatol | 0.214047 | 0.243421 | 0.260388 |
| Sulfamethoxazole | 0.354594 | 0.605785 | 0.713066 |
| Sulfisoxazole | 0.165422 | 0.32267 | 0.157768 |
| Sulforaphane | 0.180415 | 0.976828 | 2.238646 |
| Sulforaphane-N-acetylcysteine | 0.168102 | 0.168937 | 0.090055 |
| Sulfurol propionate | 0.348165 | 0.334826 | 0.327847 |
| Sulpiride | 0.701935 | 0.81657 | 0.704828 |
| Taurocholic acid | 0.312682 | 0.36701 | 0.3232 |
| Tazobactam | 0.79147 | 0.935795 | 0.813885 |
| Terbutaline | 0.152427 | 0.184747 | 0.098546 |
| terpendole K | 0.321131 | 0.542516 | 0.978596 |
| Tetradecanedioic acid | 0.17236 | 0.198547 | 0.128988 |
| Tetraglycol | 0.216286 | 1.326633 | 2.600929 |
| tetrahomomethionine | 0.28373 | 0.36305 | 0.252061 |
| Tetrahydrocortisone | 0.157787 | 0.192819 | 0.122029 |
| Tetrahydrodeoxycorticosterone | 0.157561 | 0.191159 | 0.144828 |
| tetrahydrothiophene | 0.292711 | 0.376661 | 0.396093 |
| Theobromine | 0.390348 | 0.568461 | 0.475733 |
| Theodrenaline | 0.256128 | 0.283413 | 0.292816 |
| threonylphenylalanine | 0.155584 | 0.200906 | 0.082986 |
| thymol sulfate | 0.170225 | 0.303033 | 0.146923 |
| Tiglylcarnitine | 0.143955 | 0.223793 | 0.118754 |
| Tilisolol | 0.297271 | 0.317945 | 0.285394 |
| Tixocortol pivalate | 0.546773 | 0.591653 | 0.551898 |
| Tomatidine | 0.155223 | 0.202701 | 0.116408 |
| Tranexamic Acid | 0.224919 | 0.199024 | 0.151521 |
| trans-3-Hexenoic acid | 0.176523 | 0.297176 | 0.546455 |
| trans-3-Hydroxycotinine glucuronide | 0.186818 | 0.230659 | 0.13969 |
| trans-3-Indoleacrylic acid | 0.148308 | 0.278071 | 0.138858 |
| trans-Aconitic acid | 0.497782 | 0.735006 | 0.534719 |
| trans-Cinnamaldehyde | 0.519516 | 0.567103 | 0.688743 |
| trans-Zeatin | 0.285318 | 0.419152 | 0.28294 |
| Trepibutone | 0.765782 | 0.798173 | 0.764142 |
| Triazolealanine | 0.40605 | 2.407128 | 3.400803 |
| Triethyl citrate | 0.224506 | 0.23993 | 0.181852 |
| trilobolide | 0.150284 | 0.195618 | 0.066166 |
| Trilostane | 0.225365 | 0.807984 | 1.523081 |
| Tripropionin | 0.154557 | 0.215207 | 0.10837 |
| Tris(2-chloroethyl) phosphate | 0.596277 | 3.360629 | 5.340446 |
| Troxipide | 0.171437 | 0.209572 | 0.109803 |
| trp-asn | 0.26009 | 0.318562 | 0.318903 |
| trp-gln | 1.251359 | 1.202536 | 1.410074 |
| Tyramine | 0.216763 | 0.173506 | 0.158297 |
| Tyramine-O-sulfate | 0.328387 | 0.236152 | 0.26304 |
| Tyrosylleucine | 0.171313 | 0.303673 | 0.161411 |
| Ubiquinones | 0.143381 | 0.196457 | 0.098909 |
| Undecylenic acid | 0.415197 | 0.466046 | 0.531941 |
| Uric acid | 0.384076 | 0.464273 | 0.362455 |
| Urolithin A-3-O-glucuronide | 0.233669 | 0.249989 | 0.227947 |
| Valerylglycine | 0.181983 | 1.076733 | 2.068156 |
| Valproic acid glucuronide | 0.29027 | 0.342244 | 0.292613 |
| Valyl-4-hydroxyproline | 0.173664 | 0.194153 | 0.081477 |
| Valylproline | 0.200726 | 0.375608 | 0.202452 |
| Valylvaline | 0.284595 | 0.204871 | 0.228331 |
| Viloxazine | 0.224811 | 0.241033 | 0.297489 |
| Voglibose | 0.772722 | 0.708369 | 0.723647 |
| Volkenin | 0.184812 | 0.249742 | 0.121438 |
| Xamoterol | 0.193878 | 0.177453 | 0.117697 |
| Xanthine | 0.63263 | 0.881734 | 0.675956 |
| Xanthurenic acid | 0.204458 | 0.313938 | 0.191564 |
| Zizyphine A | 0.347744 | 0.363505 | 0.397917 |
| Aspartylphenylalanine | 0.142998 | 0.219259 | 0.10682 |
| Aminobutyryl-lysine | 0.481968 | 0.830146 | 1.452028 |
| Glu-Ala | 0.310242 | 0.415253 | 0.291309 |
| Glu-Gly | 0.3239 | 0.260512 | 0.263579 |
| (-)-Caryophyllene oxide | 0.254561 | 0.264222 | 0.27309 |
| (-)-Epicatechin 7-O-glucuronide | 0.195062 | 0.210161 | 0.108122 |
| (-)-Slaframine | 0.171492 | 0.202027 | 0.120046 |
| (+)-ar-Turmerone | 1.456936 | 1.411757 | 1.404034 |
| (1R,2R,3S,6S,7R,9S,10S,11S,13R,14R)-11-(1-Hydroxy-2-propanyl)-3,7,10-trimethyl-15-oxapentacyclopentadecane-2,6,9,11,13,14-hexol | 0.347949 | 0.401549 | 0.348237 |
| 4-(hydroxymethyl)-4-cyclohexene-1,2,3-triol] | 0.229718 | 0.310433 | 0.410693 |
| (1S,3R,4R,5R)-1,3,4-trihydroxy-5-{[(2E)-3-(4-hydroxy-3-methoxyphenyl)prop-2-enoyl]oxy}cyclohexane-1-carboxylic acid | 0.157461 | 0.220712 | 0.117491 |
| (1S,4S)-menthone-8-thioacetate | 0.359417 | 0.37052 | 0.317329 |
| (1S,5R,9R,13R)-1,5,9-trimethyl-11,14,15,16-tetraoxatetracyclohexadecan-10-one | 0.547882 | 0.665347 | 0.790285 |
| (1S,5R)-5-Isopropenyl-2-methyl-2-cyclohexen-1-yl Î²-D-glucopyranoside | 0.366548 | 0.454958 | 0.452968 |
| (2E)-3-Methyl-4-(sulfooxy)-2-butenoic acid | 1.756889 | 2.583507 | 3.248098 |
| (2E)-N-(4-Amino-2-hydroxybutyl)-3-(3,4-dihydroxyphenyl)acrylamide | 0.170207 | 0.192946 | 0.114848 |
| (2R,3S,4S,5R,6S)-2-(Hydroxymethyl)-6-[4-(3-hydroxyprop-1-enyl)-2,6-dimethoxyphenoxy]oxane-3,4,5-triol | 0.158707 | 0.225907 | 0.09472 |
| (2S,3S,4R,5R)-5-(6-aminopurin-9-yl)-3,4-dihydroxyoxolane-2-carboxamide | 0.249847 | 0.187143 | 0.195592 |
| (2S,4R,9aR)-4-(2-Acetyl-4-oxo-3(4H)-quinazolinyl)-dimethyl-1,9a-dihydro-3H-spiro[furan-2,9-imidazo[1,2-a]indole]-3,5(2H,4H)-dione | 1.432741 | 1.334294 | 1.627176 |
| (2S,4R,9aS)-4-(2-Acetyl-4-oxo-3(4H)-quinazolinyl)-hydroxy-2-dimethyl-1-dihydro-3H-spiro[furan-2,9-imidazo[1,2-a]indole]-3,5(2H,4H)-dione | 0.216387 | 0.230962 | 0.149094 |
| (2S,4S)-hypoglycin B | 0.19171 | 0.369998 | 0.206855 |
| (2S,8R)-2-Amino-8-hydroxydecanoic acid | 0.170426 | 0.222746 | 0.115551 |
| (2S)-3-(1H-Imidazol-4-yl)-2-({[(3S,4S,5R)-2,3,4-trihydroxy-5-(hydroxymethyl)tetrahydro-2-furanyl]methyl}amino)propanoic acid | 0.417627 | 0.368444 | 0.376403 |
| (2S)-3-Phenyl-2-({[(3S,4S,5R)-2,3,4-trihydroxy-5-(hydroxymethyl)tetrahydro-2-furanyl]methyl}amino)propanoic acid | 0.163067 | 0.235149 | 0.090232 |
| (2S)-4-Methyl-2-({[(3S,4S,5R)-2,3,4-trihydroxy-5-(hydroxymethyl)tetrahydro-2-furanyl]methyl}amino)pentanoic acid | 0.291007 | 0.21632 | 0.238382 |
| (2Z)-12-Hydroxy-2-(hydroxymethyl)-1-methoxy-11-methyl-7-methylene-6-oxo-5,14-dioxatricyclo[9.2.1.0~4,8~]tetradec-2-en-9-yl (2Z)-2-methyl-2-butenoate | 0.554219 | 0.646187 | 0.567906 |
| (2Z)-2-(2-Ethoxy-2-oxoethylidene)succinic acid | 0.169328 | 0.198687 | 0.090592 |
| (2Z)-2-Benzylideneheptyl hydrogen sulfate | 0.465283 | 0.719191 | 1.230672 |
| (3E,5S,6S,7S,9R,11E,13E,15R,16R)-16-Ethyl-15-(hydroxymethyl)-5,7,9-trimethyl-2,10-dioxooxacyclohexadeca-3,11,13-trien-6-yl 3,4,6-trideoxy-3-(dimethylamino)-D-xylo-hexopyranoside | 0.178163 | 0.204171 | 0.225226 |
| (3R,5aS,6S,10aR)-6-Hydroxy-3-(hydroxymethyl)-2-methyl-10a-(methylsulfanyl)-3-sulfanyl-2,3,5a,6,10,10a-hexahydropyrazino[1,2-a]indole-1,4-dione | 0.217555 | 0.310916 | 0.5248 |
| (3R,5aS,6S,10aR)-6-Hydroxy-3-(hydroxymethyl)-2-methyl-3,10a-disulfanyl-2,3,5a,6,10,10a-hexahydropyrazino[1,2-a]indole-1,4-dione | 0.181181 | 0.18985 | 0.117874 |
| (3s,6R,7S)-6,7-Dihydroxy-8-methyl-8-azabicyclo[3.2.1]oct-3-yl (2E)-2-methyl-2-butenoate | 0.169978 | 0.203964 | 0.105052 |
| (3S,9S,14aR)-3,6-Dimethyl-9-{6-[(2S)-2-oxiranyl]-6-oxohexyl}decahydropyrrolo[1,2-a][1,4,7,10]tetraazacyclododecine-1,4,7,10-tetrone | 0.174336 | 0.212615 | 0.102292 |
| (3S)-3-{(Z)-[(3S)-3-{(Z)-[(3R)-3-Amino-1-hydroxy-4-methylpentylidene]amino}-1-hydroxybutylidene]amino}-5-methylhexanoic acid | 0.142592 | 0.215664 | 0.10452 |
| 4,15-Diacetoxy-3-hydroxy-12,13-epoxytrichothec-9-en-8-yl propionate | 0.155358 | 0.220004 | 0.438429 |
| (4S)-4-{[2-O-(L-Arabinofuranosyl)-L-arabinofuranosyl]oxy}proline | 0.630996 | 0.600658 | 0.607729 |
| (5Z,8Z)-5,8-Tetradecadienoic acid | 0.184557 | 0.22928 | 0.168216 |
| (6abeta)-Tazettine | 0.4766 | 0.471912 | 0.596421 |
| (6R,8Z)-6-Hydroxy-3-oxo-8-tetradecenoic acid | 0.701005 | 0.790497 | 0.743858 |
| (6R)-5-Acetamido-4-O-acetyl-3,5-dideoxy-6-[(1R)-1,2,3-trihydroxypropyl]-L-threo-hex-2-ulopyranosonic acid | 0.540722 | 0.771716 | 0.578806 |
| (8S,9Z)-9-Heptadecene-4,6-diyne-1,8-diol | 0.184325 | 0.214268 | 0.124491 |
| (Carbamoylamino)(4-hydroxyphenyl)acetic acid | 0.760532 | 0.871514 | 0.815612 |
| (E)-1,3-Tridecadiene-5,7,9,11-tetrayne | 0.372078 | 0.270637 | 0.422015 |
| (E)-3-O-Methyl entacapone | 0.328584 | 0.323197 | 0.271197 |
| (R)-1-Aminopropan-2-yl phosphate | 0.334285 | 1.032856 | 1.898265 |
| (S)-2-hydrazino-3-(4-hydroxy-3-methoxyphenyl)-2-methylpropionic acid | 0.159402 | 0.204186 | 0.109865 |
| (Z,5Z)-5-{[(1Z,2S)-6-Amino-1-{[(1Z,2R)-1-{[(1R)-1-carboxyethyl]imino}-1-hydroxy-2-propanyl]imino}-1-hydroxy-2-hexanyl]imino}-N-[(2S)-2-amino-1-hydroxypropylidene]-5-hydroxy-L-norvaline | 0.188247 | 0.228683 | 0.22372 |
| (Z)-desulfoglucotropeolin | 0.220312 | 0.330183 | 0.506384 |
| [(1S,2R,4S,5R)-5-ethenyl-1-azabicyclo[2.2.2]octan-2-yl](6-methoxyquinolin-4-yl)methanol | 0.278387 | 0.355977 | 0.277265 |
| [3-(Hydroxymethyl)-3-methyl-2-oxobicyclo[2.2.1]hept-1-yl]methyl hexopyranoside | 0.179269 | 0.253483 | 0.155587 |
| [4,6-Dihydroxy-2-methoxy-3-(3-methyl-2-buten-1-yl)phenyl]acetic acid | 0.183173 | 0.245038 | 0.150582 |
| [7-Hydroxy-1-(4-hydroxy-3-methoxyphenyl)-3-(hydroxymethyl)-6-methoxy-1,2,3,4-tetrahydro-2-naphthalenyl]methyl pentofuranoside | 0.381939 | 0.400269 | 0.381955 |
| {(1R,2R)-2-[(2Z)-5-(D-glucopyranosyloxy)pent-2-en-1-yl]-3-oxocyclopentyl}acetic acid | 0.148836 | 0.223791 | 0.116434 |
| {2-[2-(Isobutyryloxy)-4-methylphenyl]-2-oxiranyl}methyl 2-methylbutanoate | 0.154156 | 0.225723 | 0.107515 |
| 1-(3-Furyl)-1,4-pentanediol | 0.508593 | 1.430635 | 2.158423 |
| 1-(4-Hydroxy-3-methoxyphenyl)-3,5-hexadecanedione | 0.155108 | 0.251377 | 0.107449 |
| 1-(D-Ribofuranosyl)-1,3,4,7-tetrahydro-2H-1,3-diazepin-2-one | 0.312326 | 0.331567 | 0.287692 |
| 1-[(5-Amino-5-carboxypentyl)amino]-1-deoxyfructose | 0.387269 | 0.423362 | 0.597084 |
| 1-[3,4-Dihydroxy-5-(hydroxymethyl)oxolan-2-yl]-5-hydroxyimidazole-4-carboxamide | 0.469088 | 0.423151 | 0.420527 |
| 1-Adamantanamine | 0.222371 | 0.385806 | 0.567985 |
| 1-Hexyl-2-methylbenzene | 0.219908 | 0.188479 | 0.172605 |
| 1-hydroxyhexanoylglycine | 0.148515 | 0.236306 | 0.143552 |
| 1-Isothiocyanato-2-(methylthio)ethane | 0.224669 | 0.382016 | 0.659646 |
| 1-Methyladenine | 0.355671 | 0.283926 | 0.306878 |
| 1-Methylguanine | 0.184886 | 0.348895 | 0.199224 |
| 1-Methylinosine | 0.490488 | 0.436427 | 0.461799 |
| 1-Methyluric acid | 0.289896 | 0.202773 | 0.229528 |
| 1-O-3,7,12-Trihydroxy-24-oxocholan-24-yl]-D-galactopyranose | 0.231936 | 0.303844 | 0.241729 |
| 1,2-Benzisothiazolin-3-one | 0.316986 | 0.367303 | 0.322575 |
| 1,2,3,4,Tetrahydro-1,5,7-trimethylnapthalene | 0.184287 | 0.173624 | 0.090317 |
| 1,2,4-Trimethoxy-5-propenylbenzene | 0.380795 | 0.402225 | 0.522571 |
| 1,3-Dimethyluric acid | 0.15584 | 0.278945 | 0.121609 |
| 1,3,5-Heptatriene, (E,E)- | 0.491579 | 0.506304 | 0.4778 |
| 1,3,7-trimethyl-2,3,6,7-tetrahydro-1H-purine-2,6-dione | 0.142649 | 0.22335 | 0.107944 |
| 1,4-Dihydroxy-7-isopropylidene-1,4-dimethyloctahydro-6(1H)-azulenone | 0.411179 | 0.433516 | 0.380939 |
| 1,5-Anhydro-1-(2,4,6-trihydroxyphenyl)hexitol | 0.182413 | 0.191995 | 0.102707 |
| 1,5-Anhydro-1-{2-(3,4-dihydroxytetrahydro-2-furanyl)-4,7-dihydroxy-5-[(2E)-3-(4-hydroxyphenyl)-2-propenoyl]-6-oxo-6,7-dihydro-1H-indol-7-yl}hexitol | 0.703824 | 0.662692 | 0.819274 |
| 1,7-Dihydroxy-12-methyl-13-vinyl-2,10-dioxatetracyclo[5.4.1.1~8,11~.0~4,12~]tridecan-9-one | 0.199029 | 0.266665 | 0.162491 |
| 1,7-Dihydroxy-6,6-dimethyl-3,5,5a,6,7,8,9a,9b-octahydronaphtho[1,2-c]furan-9(1H)-one | 0.338206 | 0.373766 | 0.345525 |
| 1,7-Dimethyluric acid | 0.169119 | 0.315508 | 0.169573 |
| 11-Aminoundecanoic acid | 0.153241 | 0.197521 | 0.077423 |
| 17alpha-Hydroxyprogesterone | 0.31664 | 0.331576 | 0.280233 |
| 2-({6-O-[(2R,3R,4R)-3,4-Dihydroxy-4-(hydroxymethyl)tetrahydro-2-furanyl]-D-glucopyranosyl}oxy)-2-methylbutanenitrile | 0.214494 | 0.216444 | 0.155469 |
| 2-(1-Hydroxy-2,2,4,6-tetramethyl-3-oxo-2,3-dihydro-1H-inden-5-yl)ethyl hexopyranoside | 0.172566 | 0.224579 | 0.117283 |
| 2-(1,2-Dihydroxy-2-propanyl)-6,10-dimethylspiro[4.5]dec-6-en-8-one | 0.353895 | 0.406896 | 0.403242 |
| 2-(2-Carboxyethyl)-4-methyl-5-pentyl-3-furoic acid | 0.144226 | 0.223139 | 0.133948 |
| 2-(4-Methyl-5-thiazolyl)ethyl decanoate | 0.218399 | 0.237679 | 0.190798 |
| 2-(4-Methylthiazol-5-yl)ethyl butyrate | 0.300344 | 0.750074 | 2.089478 |
| 2-(6,10-Dimethyl-8-oxospiro[4.5]dec-6-en-2-yl)-2-hydroxypropyl hexopyranoside | 0.198571 | 0.252302 | 0.211591 |
| 2-(acetylamino)-3-(1H-indol-3-yl)propanoic acid | 0.149953 | 0.283641 | 0.593806 |
| 2-(Dimethylamino)-5,6-dimethylpyrimidin-4-ol | 0.252255 | 0.167045 | 0.197579 |
| 2-[4-(3-Hydroxypropyl)-2-methoxyphenoxy]-1,3-propanediol | 0.6058 | 0.809456 | 2.365916 |
| 2-Acetamido-2-deoxy-3-O-(6-deoxy-L-galactopyranosyl)-D-glucose | 0.199538 | 0.224343 | 0.140353 |
| 2-Acetamido-2-deoxy-D-galactopyranosyl-(1->4)-[6-deoxy-L-galactopyranosyl-(1->5)]-4-C-methyl-D-arabinitol | 0.242385 | 0.259127 | 0.288263 |
| 2-Acrylamido-2-methyl-1-propane sulfonic acid | 0.452646 | 0.673284 | 0.488075 |
| 2-Amino-2-deoxy-D-gluconic acid | 0.512598 | 0.563203 | 0.498325 |
| 2-Amino-3-hydroxy-3-phenylpropanoic acid | 0.209674 | 0.322165 | 0.572911 |
| 2-Amino-5-[2-(4-formylphenyl)hydrazino]-5-oxopentanoic acid | 0.273086 | 0.372795 | 0.613044 |
| 2-Amino-6-[(E)-(5-amino-5-carboxy-2-hydroxypentylidene)amino]-5-hydroxyhexanoic acid | 0.360357 | 0.271052 | 0.307605 |
| 2-Aminoadenosine | 0.157398 | 0.226053 | 0.106028 |
| 2-aminophenol sulphate | 0.484521 | 0.394291 | 0.420015 |
| 2-Butenedioic acid (2E)-, 1,4-diethyl ester | 0.177766 | 0.272696 | 0.136496 |
| 2-Butyl-5-ethyl-4-methyloxazole | 0.164809 | 0.165623 | 0.121344 |
| 2-Hexenoylcarnitine | 0.175886 | 0.160825 | 0.12889 |
| 2-Hydroxydecanedioic acid | 0.167621 | 0.223469 | 0.091037 |
| 2-Hydroxyhippuric acid | 0.154246 | 0.228926 | 0.18942 |
| 2-Isocapryloyl-3R-hydroxymethyl-butyrolactone | 0.196097 | 0.565906 | 0.966476 |
| 2-Methoxy-1,3-benzenediol | 0.273715 | 4.247725 | 5.343949 |
| 2-Methyl-3-phenylpropyl hydrogen sulfate | 0.1516 | 0.197112 | 0.079854 |
| 2-Methylbutyroylcarnitine | 0.147951 | 0.239368 | 0.122285 |
| 2-Methylhippuric acid | 0.149366 | 0.198553 | 0.257756 |
| 2-nonenoylglycine | 0.169759 | 0.186457 | 0.105281 |
| 2-octenoylglycine | 0.138892 | 0.175479 | 0.139017 |
| 2-oxo-10-methylthiodecanoic acid | 0.282385 | 0.274556 | 0.226472 |
| 2-Oxo-6-pentyltetrahydro-2H-pyran-3-carboxylic acid | 0.141398 | 0.248805 | 0.359122 |
| 2-oxo-8-methylthiooctanoic acid | 0.181049 | 0.176821 | 0.082944 |
| 2-Oxoarginine | 0.323422 | 0.260614 | 0.273092 |
| 2-Phenylethyl 6-O-D-xylopyranosyl-D-glucopyranoside | 0.183369 | 0.206561 | 0.105387 |
| 2-Phenylethyl D-glucopyranoside | 0.149423 | 0.216648 | 0.100674 |
| 2,3-Diaminosalicylic acid | 0.173798 | 0.144962 | 0.164676 |
| 2,3-Dimethoxy-5-methyl-6-(3-methyl-2-buten-1-yl)-1,4-benzenediol | 0.525022 | 0.540159 | 0.472497 |
| 2,3-Methyleneglutaric acid | 0.174119 | 0.199796 | 0.097909 |
| 2,3,4,5-tetrahydrodipicolinic acid | 0.38128 | 0.351942 | 0.345284 |
| 2,3,4,5,6-Pentahydroxy-N-(2-hydroxyethyl)hexanamide | 0.242924 | 0.329846 | 0.207426 |
| 2,3,4,9-Tetrahydro-1H-carboline-3-carboxylic acid | 0.156567 | 0.262321 | 0.126225 |
| 2,4-diacetamido-2,4,6-trideoxy-L-altrose | 0.156606 | 0.227991 | 0.142852 |
| 2,4-Diamino-6-nitrotoluene | 0.443782 | 5.477325 | 6.553687 |
| 2,4-Dihydroxy-2H-1,4-benzoxazin-3(4H)-one | 0.311841 | 0.395709 | 0.562996 |
| 2,4-Undecadien-1-al | 0.190266 | 0.199006 | 0.124952 |
| 2,8-Dihydroxyquinoline-beta-D-glucuronide | 0.196727 | 0.322235 | 0.206232 |
| 20 Dihydrocortisol | 0.18455 | 0.192592 | 0.12144 |
| 3-(1-carboxyvinyloxy)anthranilic acid | 0.150293 | 0.2165 | 0.105591 |
| 3-(2-methylpropyl)-octahydropyrrolo[1,2-a]pyrazine-1,4-dione | 0.136496 | 0.192154 | 0.138914 |
| 3-(3,4-Dimethoxyphenyl)-2-propenoic acid | 0.161798 | 0.188551 | 0.08955 |
| 3-(6-Amino-1H-purin-1-yl)-1-propanol | 0.165556 | 0.192334 | 0.094933 |
| 3-(6-hydroxyindol-3-yl)lactic acid | 0.145131 | 0.200803 | 0.072638 |
| 3-(Hydroxymethyl)-1-oxo-1H-isochromen-6-yl hexopyranosiduronic acid | 0.279528 | 0.388844 | 0.585382 |
| 3-[(1S)-2-Cyclohexen-1-yl]-L-alanine | 0.125672 | 1.088026 | 2.109898 |
| 3-[(2-Carboxy-2-hydroxyethyl)dithio]-L-alanine | 0.376044 | 0.327648 | 0.326819 |
| 3-[(2Z)-1-Hydroxy-2-buten-2-yl]pentanedioic acid | 0.152326 | 0.212775 | 0.15662 |
| 3-[(3-Hydroxyheptanoyl)oxy]-4-(trimethylammonio)butanoate | 0.162271 | 0.25487 | 0.115731 |
| 3-[(3-Hydroxynonanoyl)oxy]-4-(trimethylammonio)butanoate | 0.171243 | 0.238181 | 0.121037 |
| 3-[(4-hydroxyphenyl)methyl]-octahydropyrrolo[1,2-a]pyrazine-1,4-dione | 0.381629 | 0.582612 | 0.763403 |
| 3-[(6-Oxodecanoyl)oxy]-4-(trimethylammonio)butanoate | 0.793184 | 0.697618 | 0.777755 |
| 3-{[(2E)-4-Methoxy-4-oxo-2-butenoyl]amino}-L-alanyl-L-leucine | 0.159157 | 0.20074 | 0.110046 |
| 3-Acetyl-6-hydroxy-4a,5-dimethyl-4a,5,6,7,8,8a-hexahydro-2(1H)-naphthalenone | 0.292538 | 0.530848 | 0.75668 |
| 3-Hydroxycarbofuran | 0.151437 | 0.186843 | 0.08417 |
| 3-hydroxydecanoyl carnitine | 0.174724 | 0.219724 | 0.117799 |
| 3-Hydroxydodecanedioic acid | 0.153818 | 0.222291 | 0.095541 |
| 3-hydroxydodecanoyl carnitine | 0.255821 | 0.261539 | 0.192456 |
| 3-Hydroxyhexanoylcarnitine | 0.148089 | 0.275076 | 0.150865 |
| 3-hydroxyoctanoyl carnitine | 0.289159 | 0.336023 | 0.272159 |
| 3-Hydroxysebacic acid | 0.163365 | 0.281898 | 0.144306 |
| 3-Hydroxytetradecanedioic acid | 0.154293 | 0.218632 | 0.105431 |
| 3-Ketocarbofuran | 0.16722 | 0.19949 | 0.091528 |
| 3-Mercaptohexyl butyrate | 0.314869 | 0.212759 | 0.261729 |
| 3-Methoxyestra-1,3,5(10),16-tetraene | 0.171769 | 0.176199 | 0.102384 |
| 3-Methoxytyrosine | 0.387678 | 0.334298 | 0.372071 |
| 3-Methylcrotonylglycine | 0.152271 | 0.256121 | 0.196175 |
| 3-Methylglutarylcarnitine | 0.224688 | 0.389234 | 0.235568 |
| 3-Methylhistamine | 0.378316 | 0.301151 | 0.450531 |
| 3-O-(L-olivosyl)oleandolide | 0.35047 | 0.377737 | 0.387308 |
| 3-oxo-C12-HSL | 0.751008 | 0.686687 | 0.842353 |
| 3-Oxoglutaric acid | 0.548103 | 0.797772 | 0.58469 |
| 3-Oxotetradecanoic acid | 0.17157 | 0.164323 | 0.092652 |
| 3-Phenylpent-4-enal | 0.267103 | 0.282215 | 0.295089 |
| 3-Ureidopropionic acid | 0.388325 | 0.326225 | 0.330726 |
| 3,10-Diamino-14-methyl-5,6,6a,6b,7,9-hexahydro-4H-pyrimido[1,4]diazepinopyrrolo[1,2-f]pteridine-1,12-dione | 1.283515 | 1.315622 | 1.337591 |
| 3,12,13-Trihydroxy-11-methyl-6-methylene-16-oxo-15-oxapentacycloheptadecane-9-carboxylic acid | 0.385887 | 0.535815 | 0.48349 |
| 3,4-Dimethyl-5-pentyl-2-furanpropanoic acid | 0.428036 | 0.428799 | 0.398135 |
| 3,4-Methyleneazelaic acid | 0.167778 | 0.454175 | 0.8604 |
| 3,4-Methylenesebacic acid | 0.490227 | 0.528759 | 0.48135 |
| 3,7-Dimethyl-2,6-octadienal | 0.337801 | 0.581575 | 1.107551 |
| 3'-Amino-3'-deoxythimidine glucuronide | 0.174598 | 0.233698 | 0.140336 |
| 3',5,7-Trihydroxy-4'-methoxyflavanone | 0.182911 | 0.17229 | 0.131611 |
| 4-(1-Carboxyethyl)-3-hydroxy-2-(3-methyl-2-buten-1-yl)phenyl hexopyranosiduronic acid | 0.227434 | 0.222286 | 0.204948 |
| 4-(5-Hydroxy-2-methyl-2-azabicyclo[2.2.2]oct-5-yl)-3-methylbutanoic acid | 0.307806 | 0.327221 | 0.309732 |
| 4-[(2-Isopropyl-5-methylcyclohexyl)oxy]-4-oxobutanoic acid | 0.383146 | 0.439739 | 0.392837 |
| 4-[(2-Methyl-3-furyl)thio]-5-nonanone | 0.299962 | 0.388813 | 0.287204 |
| 4-[2-Amino-3-(D-glucopyranosyloxy)phenyl]-4-oxobutanoic acid | 0.407868 | 0.413303 | 0.607887 |
| 4-Acetamidobutanoic acid | 0.272663 | 0.144693 | 0.216044 |
| 4-Acetoxy-2-hexyltetrahydrofuran | 0.250128 | 0.396294 | 0.536649 |
| 4-Aminohippuric acid | 0.223261 | 0.401759 | 0.245998 |
| 4-Guanidinobutyric acid | 0.260028 | 0.186348 | 0.205836 |
| 4-hydroxy-4-(indol-3-ylmethyl)glutamic acid | 0.153439 | 0.216014 | 0.096546 |
| 4-Hydroxy-5-(2-hydroxy-2-propanyl)-2-methylbicyclo[3.1.0]hex-2-yl hexopyranoside | 0.159323 | 0.227224 | 0.107927 |
| 4-Hydroxy-5-methoxy-4-[2-methyl-3-(3-methylbut-2-en-1-yl)oxiran-2-yl]-1-oxaspiro[2.5]octan-6-one | 0.415667 | 0.494686 | 0.427246 |
| 4-Hydroxy-8-(D-talopyranosyloxy)-2-quinolinecarboxylic acid | 1.194884 | 1.290445 | 1.196596 |
| 4-Hydroxycyclohexylcarboxylic acid | 0.330318 | 0.842766 | 2.403774 |
| 4-Hydroxyprolyltryptophan | 0.21241 | 0.320296 | 0.355715 |
| 4-Imino-1-(D-ribofuranosyl)-1,4-dihydro-3-pyridinecarboxylic acid | 0.626393 | 0.58948 | 0.605776 |
| 4-Methoxycinnamaldehyde | 0.156663 | 0.179693 | 0.083338 |
| 4-Methyl-2-propyltetrahydro-2H-pyran-4-yl acetate | 0.270894 | 0.253541 | 0.296031 |
| 4-Methylesculetin | 0.170987 | 0.200403 | 0.095914 |
| 4-Methylumbelliferone hydrate | 0.181344 | 0.168817 | 0.114255 |
| 4-O-(4-Deoxy-L-threo-hex-4-enopyranuronosyl)-D-galactopyranuronic acid | 0.168893 | 0.258251 | 0.123337 |
| 4-O-(L-Araf)-cis-L-Hyp | 0.395347 | 0.54945 | 0.407396 |
| 4-O-D-Glucopyranosylmoranoline | 0.198436 | 0.303968 | 0.165604 |
| 4-Octylphenol | 0.169418 | 0.195086 | 0.113232 |
| 4-Phenyl-3-buten-2-one | 0.253315 | 0.292761 | 0.421501 |
| 4-Phenylbutyric acid | 0.169971 | 0.178328 | 0.22612 |
| 4-Pyridoxic acid | 0.289085 | 0.18074 | 0.236838 |
| 4-Trimethylammoniobutanoic acid | 0.378977 | 0.33956 | 0.333835 |
| 4,4-Thiobis(2-butanone) | 0.156203 | 0.219842 | 0.114154 |
| 4,5-Dihydroxy-3-oxo-1-cyclohexene-1-carboxylic acid | 0.306024 | 0.483998 | 0.334126 |
| 4,7-Dimethoxy-3-oxo-3,4-dihydro-2H-1,4-benzoxazin-2-yl D-glucopyranoside | 0.206043 | 0.22405 | 0.1391 |
| 4'-Methoxyacetophenone | 0.180561 | 0.168285 | 0.115596 |
| 4a,5-dihydroriboflavin | 0.292451 | 0.376518 | 0.296891 |
| 5-(2,3-Dihydroxy-3-methylbutyl)-4-[(3,3-dimethyl-2-oxiranyl)acetyl]-3,4-dihydroxy-2-(3-methylbutanoyl)-2-cyclopenten-1-one | 0.340216 | 0.425656 | 0.33139 |
| 5-(3',4'-Dihydroxyphenyl)-gamma-valerolactone-3'-O-methyl-4'-O-glucuronide | 0.20333 | 0.276096 | 0.242057 |
| 5-[(Z)-(4-Ethyl-3-methyl-5-oxo-1,5-dihydro-2H-pyrrol-2-ylidene)methyl]-5-methoxy-3-methyl-4-vinyl-1,5-dihydro-2H-pyrrol-2-one | 0.171866 | 0.184286 | 0.116829 |
| 5-Acetylamino-6-amino-3-methyluracil | 0.338826 | 0.265549 | 0.320089 |
| 5-aminosalicyluric acid | 0.412906 | 0.364822 | 0.356586 |
| 5-Butyloxazole | 0.146182 | 0.244515 | 0.110065 |
| 5-Hydantoinpropionic acid | 0.382711 | 0.514114 | 0.375364 |
| 5-Hydroxy-3-(4-hydroxyphenyl)-6-methoxy-7-[(2S,4S,5S)-3,4,5-trihydroxy-6-(hydroxymethyl)oxan-2-yl]oxychromen-4-one | 0.206416 | 0.238639 | 0.194096 |
| 5-Hydroxy-L-tryptophan | 0.205894 | 0.157593 | 0.151588 |
| 5-Hydroxyindole-3-acetic acid | 0.152693 | 0.21621 | 0.122772 |
| 5-Hydroxyindoleacetaldehyde | 0.4765 | 4.849618 | 5.860421 |
| 5-Hydroxyomeprazole | 0.159076 | 0.226776 | 0.115586 |
| 5-Methoxybenzimidazole | 0.361537 | 0.512448 | 0.500621 |
| 5-Methylangelicin | 0.315496 | 0.261319 | 0.267641 |
| 5-Methyltetrahydrofolic acid | 0.40529 | 0.457839 | 0.372005 |
| 5-methylthioribose | 0.394543 | 0.344147 | 0.356216 |
| 5-Nitro-o-toluidine | 0.298825 | 0.169064 | 0.227641 |
| 5-Sulfanyl-L-histidine | 0.216084 | 0.268094 | 0.167938 |
| 5,5-Dihydroxy-tetramethyl-4,5-dihydro-2H,3H-spiro[furan-2,6-[7]oxabicyclo[3.2.1]oct[3]en]-one | 0.137773 | 0.216802 | 0.091906 |
| 5,7,11-Trihydroxy-7-(methoxymethyl)-2-methyl-10H-spiro[9-oxatricyclo[6.3.1.0~1,5]dodecane-6,3oxetane]10-dione | 0.214861 | 0.269513 | 0.148408 |
| 5,8,12-Trihydroxy-2-oxododecanoic acid | 0.188282 | 0.239948 | 0.117795 |
| 5'-O-beta-D-Glucosylpyridoxine | 0.187962 | 0.2812 | 0.133289 |
| 6 Hydroxycortisol | 0.145399 | 0.207275 | 0.090997 |
| 6-[(1E)-3,4-Dihydroxy-3-methyl-1-buten-1-yl]-7-methoxy-2H-chromen-2-one | 0.287639 | 0.334916 | 0.344308 |
| 6-[(1R,2S)-1,2-Dihydroxypropyl]-3,4-dihydro-2,4-pteridinediol | 0.233104 | 0.153092 | 0.176159 |
| 6-Amino-2-methyl-2-heptanyl (1,3-dimethyl-2,6-dioxo-1,2,3,6-tetrahydro-7H-purin-7-yl)acetate | 0.33222 | 0.393949 | 0.350302 |
| 6-Hydroxy-5-methoxyindole glucuronide | 0.151974 | 0.208701 | 0.097173 |
| 6-Hydroxy-5-methyl-4,11-dioxoundecanoic acid | 0.375479 | 1.894208 | 3.091779 |
| 6-Hydroxy-8-methyl-8-azabicyclo[3.2.1]octan-3-yl 3-hydroxy-2-phenylpropanoate | 0.180543 | 0.23578 | 0.149031 |
| 6-Hydroxypentadecanedioic acid | 0.519186 | 0.580059 | 0.593053 |
| 6-Sulfatoxymelatonin | 0.216703 | 0.374362 | 0.203563 |
| 6-Thiouric acid | 0.406417 | 0.576783 | 0.424949 |
| 7-(3-Amino-3-carboxypropyl)-4,6-dimethyl-3-(D-ribofuranosyl)-3,4-dihydro-9H-imidazo[1,2-a]purin-9-one | 0.287992 | 0.29128 | 0.261668 |
| 7-[1-Formyl-6-hydroxy-6-(hydroxymethyl)bicyclo[3.2.1]oct-2-yl]-3a,7-dimethyl-3-oxooctahydro-2-benzofuran-1-yl hexopyranoside | 0.285725 | 0.360454 | 0.267243 |
| 7-Isopropyl-4a-methyl-1-methylene-1,2,3,4,4a,9,10,10a-octahydrophenanthrene | 0.271392 | 0.287626 | 0.247027 |
| 7-Mercaptoheptanoylthreonine | 0.890246 | 0.925024 | 0.890357 |
| 7-Methylxanthine | 0.211842 | 0.2442 | 0.138329 |
| 7,8-Dihydrobiopterin | 0.359871 | 0.582503 | 0.401795 |
| 7alpha,17beta-Dihydroxyandrost-4-en-3-one | 0.227127 | 0.224643 | 0.237475 |
| 7C-aglycone | 0.165707 | 0.19427 | 0.103244 |
| 8-(3-Furyl)-5-hydroxy-1,1,5a,7a,11b-pentamethyldecahydrooxireno[4,4a]isochromeno[6,5-g][2]benzoxepine-3,10,12(1H,4H,10aH)-trione | 0.34269 | 0.393447 | 0.417367 |
| 8-{[2-Hydroxy-2-(4-hydroxyphenyl)ethyl](methyl)amino}-1,3,7-trimethyl-3,7-dihydro-1H-purine-2,6-dione | 0.19955 | 0.254938 | 0.12742 |
| 8-Epideoxyloganic acid | 0.183087 | 0.227335 | 0.138661 |
| 8-Hydroxy-4-methoxy-7-methyl-7,8-dihydro-5H-furo[2,3-g]isochromen-5-one | 0.179834 | 0.245997 | 0.144333 |
| 8-Hydroxy-5,6-octadienoic acid | 0.165926 | 0.698742 | 1.209775 |
| 8,8a-Diepiswainsonine | 0.152113 | 0.231649 | 0.074541 |
| 9-(D-glucosyl)dihydrozeatin | 0.221436 | 0.21907 | 0.169796 |
| 9-Decenoylcarnitine | 0.153543 | 0.175939 | 0.129682 |
| 9-Methyluric acid | 0.58836 | 0.538194 | 0.552006 |
| 9-O-Demethyl-2-hydroxyhomolycorine | 0.438753 | 0.428139 | 0.416089 |
| 9,10,13-TriHOME | 0.084229 | 3.084001 | 4.752036 |
| 9,12,13-TriHOME | 0.076221 | 2.964374 | 4.577394 |

**Figure S16**. Analytical stability of the acquisition monitored by the ¹⁵N₂-tryptophan internal standard, which was spiked at a constant amount into every injection and therefore carries no biological variation. (A) ¹⁵N₂-tryptophan peak area as a function of injection order across the full acquisition; the grey band marks the mean ± the overall relative standard deviation (RSD). Signal was stable across the run with negligible drift (under 2% change in mean signal from the first to the last third of the injections). (B) ¹⁵N₂-tryptophan RSD for each clinical cohort, for the pooled QC injections, and overall. Reproducibility was comparable in clinical and QC injections (clinical 14.3%, QC 14.6%, total 14.4%; range 8.4 to 15.0% across cohorts), indicating a stable platform and a matrix-independent internal-standard response. This analytical variability is well below the biological variability of urinary creatinine (Figure S17), confirming that the poor performance of creatinine normalisation reflects biological variation in creatinine excretion rather than instrument drift or matrix effects. Values of n are injections: NIP 107, iPENS 862, CD-Treat 125, pooled QC 250.

**Figure S17**. Coefficient of variation (CV) of the urinary creatinine signal (peak area) across the clinical cohorts, illustrating why creatinine is an unstable normalisation factor in this population. (A) Distribution of the creatinine peak area for each clinical group and for the pooled quality control (QC) samples; boxes show the median and interquartile range, whiskers the 1.5× interquartile range, and points individual injections. (B) Creatinine signal CV per group; the dashed line marks the analytical CV of the pooled QC samples (3.0%). Biological variability in creatinine excretion (CV 24 to 35% across clinical groups) exceeds the analytical variability of the creatinine measurement by more than ten-fold, and is of similar magnitude in Crohn’s disease and healthy control samples. iPENS Crohn’s n=808, iPENS healthy n=54, CD-TREAT paediatric n=61, CD-TREAT adult n=58, pooled QC n=194.
